# CpG‐Based Nanovaccines Enhance Ovarian Cancer Immune Response by Gbp2‐Mediated Remodeling of Tumor‐Associated Macrophages

**DOI:** 10.1002/advs.202412881

**Published:** 2025-02-22

**Authors:** Jiaqiang Xiong, Juyuan Huang, Hanxiao Xu, Qiuji Wu, Jiahui Zhao, Yurou Chen, Guanlan Fan, Haotong Guan, Rourou Xiao, Zhaojin He, Siqi Wu, Wenliang Ouyang, Shixuan Wang, Lu Zhang, Peng Xia, Wei Zhang, Meng Wu

**Affiliations:** ^1^ Department of Obstetrics and Gynecology Zhongnan Hospital of Wuhan University Wuhan 430071 China; ^2^ Department of Gastrointestinal Oncology Zhongnan Hospital of Wuhan University Wuhan 430071 China; ^3^ Department of Radiation and Medical Oncology Hubei Key Laboratory of Tumor Biological Behavior Hubei Provincial Clinical Research Center for Cancer Zhongnan Hospital of Wuhan University Wuhan 430071 China; ^4^ The Second Clinical College of Wuhan University Wuhan 430071 China; ^5^ Department of Obstetrics and Gynecology Tongji Hospital Tongji Medical College Huazhong University of Science and Technology Wuhan 430032 China; ^6^ Hubei Key Laboratory of Radiation Chemistry and Functional Materials School of Nuclear Technology and Chemistry & Biology Hubei University of Science and Technology Xianning 437100 China; ^7^ Department of Hepatobiliary & Pancreatic Surgery Zhongnan Hospital of Wuhan University Wuhan 430071 China

**Keywords:** Gbp2, immunotherapy, nanovaccine, ovarian cancer, TAMs

## Abstract

CpG oligodeoxynucleotides (CpG), as an immunoadjuvant, can facilitate the transformation of tumor‐associated macrophages (TAMs)into tumoricidal M1 macrophages. However, the accumulation of free CpG in tumor tissues remains a substantial challenge. To address this, a nanovaccine (PLGA‐CpG@ID8‐M) is engineered by encapsulating CpG within PLGA using ID8 ovarian cancer cell membranes (ID8‐M). This nanovaccine demonstrates remarkable efficacy in reprogramming TAMs in ovarian cancer and significantly extends survival in ID8‐bearing mice. Notably, these findings indicate that the nanovaccine can also mitigate chemotherapy‐induced immunosuppression by increasing the proportion of M1‐like TAMs and reducing the expression of CD47 on tumor cells, thereby achieving a synergistic effect in tumor immunotherapy. Mechanistically, through transcriptome sequencing (RNA‐seq), single‐cell RNA sequencing (scRNA‐seq), and mass spectrometry‐based proteomics, it is elucidated that the nanovaccine enhances the expression of Gbp2 and promotes the recruitment of Pin1, which activates the NFκB signaling pathway, leading to the M1 polarization of TAMs. Furthermore, macrophages with elevated Gbp2 expression significantly inhibit tumor growth in both ID8 ovarian cancer and 4T1 breast cancer models. Conversely, targeting Gbp2 diminishes the antitumor efficacy of the nanovaccine in vivo. This study offers an innovative approach to immunotherapy and elucidates a novel mechanism (Gbp2‐Pin1‐NFκB pathway) for remodeling TAMs.

## Graphical abstract

CpG‐based nanovaccines bolster ovarian cancer immunotherapy via the Gbp2‐Pin1‐NFkB pathway‐mediated reprogramming of tumor‐associated macrophages (TAMs.) Moreover, this nanovaccine can alleviate chemotherapy‐induced immunosuppression by augmenting the proportion of M1‐like TAMs, enhancing the infiltration of CD8^+^ T cells, and downregulating the expression of CD47 through the reduction of HIF1A in ID8 tumor cells. Therefore, it achieves synergistic anti‐ovarian cancer effect with chemotherapy.

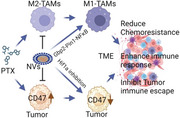

## 1 Introduction

Ovarian cancer is the most lethal gynecological malignancy, with a 5‐year survival rate for advanced patients falling below 30%.^[^ ^1^ ^]^ The poor prognosis associated with ovarian cancer can be attributed to its resistance to chemotherapy as well as a lower response rate to immunotherapy largely due to the immunosuppressive tumor microenvironment (TME).^[^ ^2^, ^3^, ^4^ ^]^ TAMs, which can comprise up to 30%–50% of the immune cells in ovarian cancers and predominantly exhibit an M2 phenotype, play a pivotal role in creating this inhibitory immune environment.^[^ ^5^, ^6^ ^]^ Furthermore, TAMs contribute to chemoresistance, further complicating patient outcomes.^[^ ^7^, ^8^ ^]^ Approximately 80% of patients with advanced ovarian cancer eventually develop resistance to chemotherapy, a phenomenon closely linked to the presence of TAMs.^[^ ^9^, ^10^, ^11^ ^]^ Therefore, reprogramming TAMs to adopt an M1 phenotype not only alleviates the immunosuppressive conditions in ovarian cancer but also helps overcome tumor chemoresistance. In clinical studies, targeting TAMs through the activation of toll‐like receptors (TLRs) has emerged as one of the most extensively investigated strategies for stimulating adaptive immune response. Imiquimod (R837), one of the first compounds in this category, demonstrated promising results in clinical trials for basal cell carcinoma and received Food and Drug Administration (FDA) approval in 2004.^[^ ^12^ ^]^ Following the success of imiquimod, other drugs such as resiquimod (R848) and 852‐A were developed for systemic treatment of metastatic cancers, including colon, breast, ovarian, and cervical cancers.^[^ ^13^, ^14^ ^]^ These agents showed potential in stabilizing disease in some patients. However, the systemic induction of cytokines associated with these treatments often led to severe side effects, resulting in the premature termination of several clinical trials.^[^ ^15^, ^16^ ^]^ Consequently, due to the limited targeting ability and considerable toxic side effects associated with many free TAM‐targeting drugs, it is imperative to explore more effective strategies for targeting TAMs.

In recent years, nanovaccines—primarily composed of tumor antigens and immunoadjuvants—have emerged as promising tools in tumor immunotherapy due to their targeted delivery capabilities.^[^ ^17^, ^18^ ^]^ For example, a nanovaccine derived from Escherichia coli cytoplasmic membranes and tumor cell membranes, extracted from resected autologous tumor tissue, has been shown to induce dendritic cells (DCs) maturation and activate T cells in the CT26 colon and 4T1 breast tumor mouse models.^[^ ^19^ ^]^ Additionally, nanovaccines utilizing virus‐like particles derived from hepatitis B core antigen (HBcAg) have shown remarkable antitumor efficacy by effectively promoting T cell infiltration and repolarizing M2‐like TAMs in melanoma.^[^ ^20^ ^]^ While several studies have investigated nanovaccines targeting DCs in peripheral lymph nodes or tumor tissues in ovarian cancer,^[^ ^21^, ^22^ ^]^ few have explored nanovaccines specifically targeting TAMs. Given that TAMs are the predominant immune cells in ovarian cancer and play a critical role in tumor progression and prognosis, it is essential to investigate the potential clinical significance of nanovaccines in reshaping TAMs and elucidating their antitumor mechanisms in this context.

CpG, a synthetic 18–30 bp non‐methylated cytosine‐guanine dinucleotide DNA sequence, is an FDA‐approved immunoadjuvant that promotes the transformation of TAMs into tumor‐killing M1 phenotypes.^[^ ^23^ ^]^ However, the short half‐life and negative charge of CpG hinder its accumulation in tumor tissues when administered in free form. Clinical studies have indicated that moderate doses of CpG result in insufficient antitumor activity, while higher doses are associated with frequent adverse events.^[^ ^24^ ^]^ Therefore, developing strategies for the precise delivery of CpG is essential to mitigate the toxicity associated with high doses, thereby enhancing its clinical applicability. Tumor cell membranes, as effective drug carriers, retain full tumor cell membrane antigens, which can enhance vaccine efficacy. Furthermore, tumor cell membrane‐coated nanovaccines can prolong circulation time, improve tumor antigen presentation, and increase targeting precision. Consequently, utilizing nanovaccines composed of tumor cell membrane‐associated CpG immunoadjuvants to modulate TAM polarization towards the M1 phenotype presents a promising approach.

In the present study, we utilized FDA‐approved biodegradable PLGA loaded with CpG immunoadjuvant,^[^ ^25^ ^]^ isolated ID8‐M, and coated these onto the surface of the PLGA to develop the PLGA‐CpG@ID8‐M nanovaccine. This nanovaccine is designed to specifically target ovarian cancer, facilitating the phagocytosis and remodeling of TAMs both in vivo and in vitro. Notably, nanovaccines engulfed by ID8 cells can reduce CD47 expression without compromising cell proliferation, thereby enhancing macrophage phagocytosis by attenuating the CD47‐SIRPα inhibitory signaling.^[^ ^26^ ^]^ In the ID8 tumor‐bearing mouse model, our findings indicate that the nanovaccine can reverse the paclitaxel‐induced increase in M2‐like TAMs, achieving a synergistic anti‐ovarian cancer effect when combined with chemotherapy. To elucidate the mechanisms by which nanovaccine reshapes TAMs, we utilized RNA‐seq and scRNA‐seq, supplemented with biological validation. Our results reveal, for the first time, that PLGA‐CpG@ID8‐M nanovaccine facilitates the shift of TAMs towards an antitumor M1 phenotype through the Gbp2‐Pin1‐NFκB signaling axis. This study pioneers the exploration of the role of nanovaccine in facilitating TAM polarization, delineating the underlying mechanisms and establishing a robust theoretical framework for the deployment of nanovaccine in ovarian cancer treatment.

## 2 Results and Discussion

### 2.1 Synthesis, Characterization, and Safety Assessment of PLGA‐CpG@ID8‐M Nanovaccine

**Figure 1:**
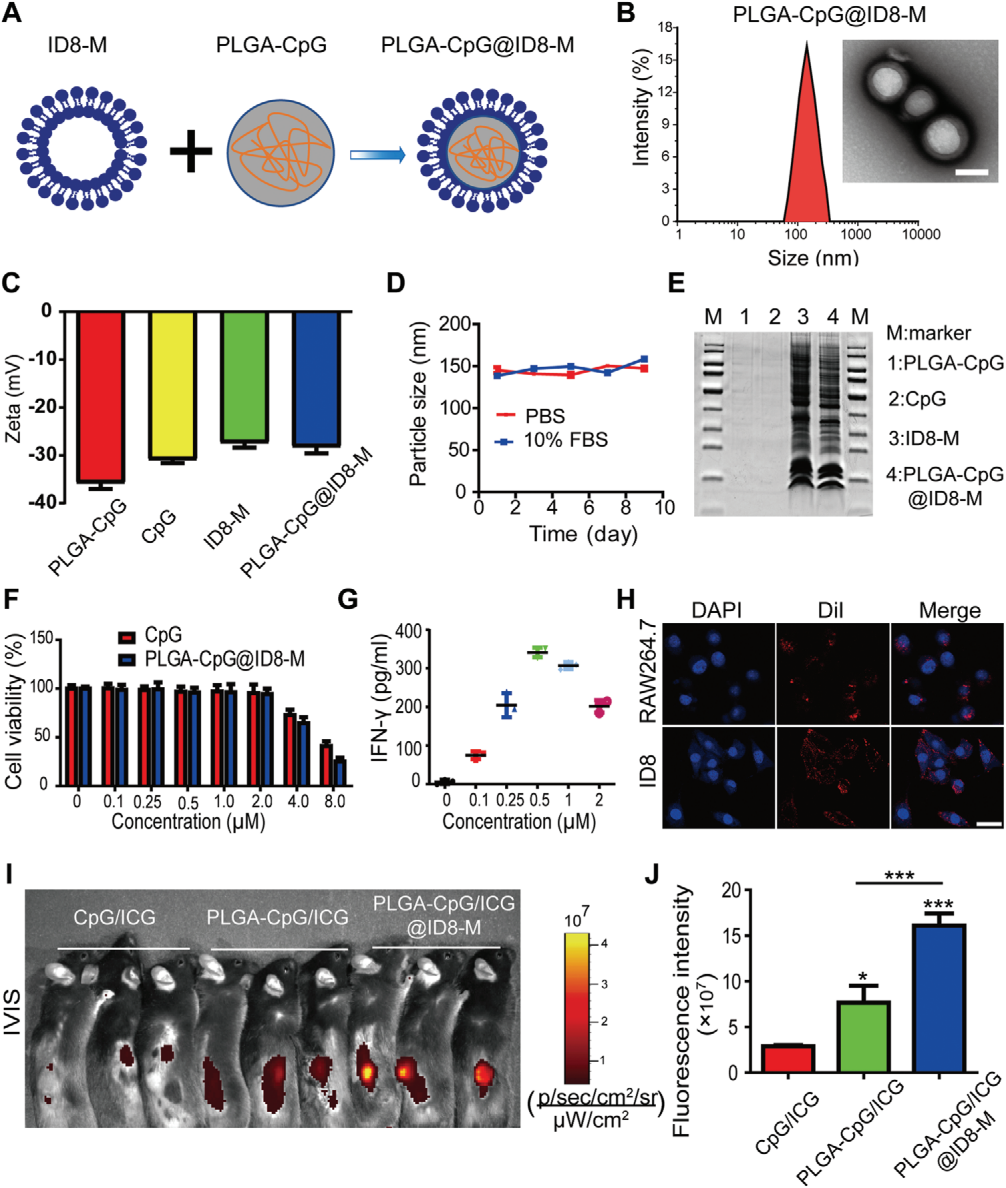
Synthesis and safety evaluation of nanovaccines. A) Schematic illustration of the synthesis of PLGA‐CpG@ID8‐M nanovaccine. B) Size distribution and TEM images of PLGA‐CpG@ID8‐M (Scale bar: 100 nm). C) Surface zeta potential measurements of PLGA‐CpG, CpG, ID8‐M, and PLGA‐CpG@ID8‐M. D) Long‐term stability of PLGA‐CpG@ID8‐M in PBS and 10% FBS. E) SDS‐PAGE protein analysis of PLGA‐CpG, CpG, ID8‐M, and PLGA‐CpG@ID8‐M using Coomassie blue staining. F) Cell viability of RAW264.7 macrophages incubated with various concentrations of PLGA‐CpG@ID8‐M for 24 h. G) Secretion of IFN‐γ detected by ELISA after BMDMs were cocultured with the nanovaccine for 24 h. H) CLSM images of ID8 and RAW264.7 cells incubated with DiI‐labeled PLGA‐CpG@ID8‐M for 4 h, scale bars: 20 µm. I,J) Tumor‐targeting capability of the nanovaccine (1 nmol of CpG per mouse) assessed by IVIS following intravenous injection into ID8 tumor‐bearing mice for 6 h (*n* = 3). Data are presented as mean ± SD, analyzed using an unpaired two‐sided Student's t test (*p < 0.05, **p < 0.01, ***p < 0.001).

To fabricate the nanovaccine, we synthesized the PLGA‐CpG nanoparticles using the ultrasound double emulsion technique, followed by encapsulating ID8‐M through a liposome extrusion process, similar to our previous methods.^[^ ^17^ ^]^ This resulted in the final synthesis of the PLGA‐CpG@ID8‐M nanovaccine (**Figure** **1**A). Through dynamic light scattering (DLS) detection, the average hydrated particle size of the nanovaccine was 144 nm, and the average diameter of nanovaccine was 115 nm under transmission electron microscope (TEM) (Figure 1B). Furthermore, the surface zeta potential of the nanovaccine was approximately ‐28.3 mV (Figure 1C). In addition, the long‐term stability of PLGA‐CpG@ID8‐M in phosphate buffer saline (PBS) and 10% fetal bovine serum (FBS) was evaluated by DLS, showing consistent nanoparticle size over 9 d, indicating high stability (Figure 1D). Protein analysis via coomassie blue staining revealed similar membrane protein bands between the nanovaccine and free ID8‐M, suggesting that the membrane proteins remained stable during synthesis (Figure 1E). Notably, the loading efficiency of CpG was 92%, demonstrating excellent encapsulation via the ultrasound double emulsion technique (Figure S1, Supporting Information).

To evaluate the cytotoxicity of the nanovaccine in vitro, we cocultured PLGA‐CpG@ID8‐M with RAW264.7 macrophages at various CpG concentrations in 96‐well plates, assessing cell proliferation using Cell Counting Kit‐8 (CCK8) assay. Neither PLGA‐CpG@ID8‐M nor free CpG significantly affected cell viability at concentrations of 0–2 µm, while obvious cytotoxicity was observed at concentrations exceeding 4 µm (Figure 1F). To assess the immunostimulatory effect of PLGA‐CpG@ID8‐M within a safe concentration range, varying concentrations were cocultured with bone marrow‐derived macrophages (BMDMs) for 24 h. Enzyme‐Linked Immunosorbent Assay (ELISA) analysis indicated that a 0.5 µm concentration of the nanovaccine elicited the highest secretion of IFN‐γ (Figure 1G). In addition, to observe the long‐term cytotoxic effects of both free CpG and PLGA‐CpG@ID8‐M (0.5 µm) on BMDMs over a 96 h period, the results showed that the 0.5 µm nanovaccine concentration exhibited no cytotoxic effects, confirming its safety (Figure S2, Supporting Information). To further investigate the cytotoxic effects of the nanovaccine on other cell types at this concentration, RAW264.7, ID8, 4T1, HeLa, and 3T3 cells were tested, with results showing no cytotoxicity (Figure S3, Supporting Information). Therefore, a nanovaccine concentration of 0.5 µm was selected for further experiment in vitro. To visualize phagocytosis, 1,1′‐dioctadecyl‐3,3,3′,3′‐tetramethylindocarbocyanine perchlorate (DiI)‐labeled nanovaccines were incubated with RAW264.7 and ID8 cells for 4 h. Confocal laser scanning microscopy (CLSM) revealed rapid uptake of the nanovaccine by both macrophages and tumor cells (Figure 1H). Additionally, to evaluate in vivo tumor‐targeting capability, ICG‐encapsulated nanovaccines were administered intravenously to ID8 tumor‐bearing mice. Our results demonstrated that the ID8‐M‐coated nanovaccines showed significantly enhanced accumulation in tumor tissues compared to free ICG and PLGA‐ICG nanoparticles, as detected by an in vivo imaging system (IVIS) (Figure 1I,J). Furthermore, to indicate the percentage of nanovaccine reaching tumor sites, tumor tissue was dissected, weighed and homogenized in DMSO solution to extract ICG, and the concentrations of ICG were determined by fluorometry and presented as percentage injected dose per gram of tissue (ID g^−1^). The results indicated that approximately 15% of the nanovaccine successfully reached the tumor site. In contrast, less than 1% of the free ICG accumulated at the tumor site, and only about 4% of the nanoparticles without the ID8‐M coating were able to reach the tumor tissue (Figure S4, Supporting Information). To eliminate potential confounding effects from the ID8 tumor during the biosafety evaluation, wild‐type 8‐week‐old female C57BL/6 mice were randomly divided into two groups (*n* = 5), receiving either PBS or the nanovaccine via tail vein injection on days D1, D5, and D9. Body weights were monitored, and histological examinations, along with biochemical assays of major organs (heart, liver, spleen, lungs, kidneys) were performed (Figure S5, Supporting Information). Comparative analysis with the PBS group revealed no significant differences, indicating a favorable safety profile for the nanovaccine in vivo.

### 2.2 Nanovaccines Induce Antitumor Effects by Enhancing M1 Polarization of TAMs and Reducing CD47‐Mediated Immune Evasion

**Figure 2:**
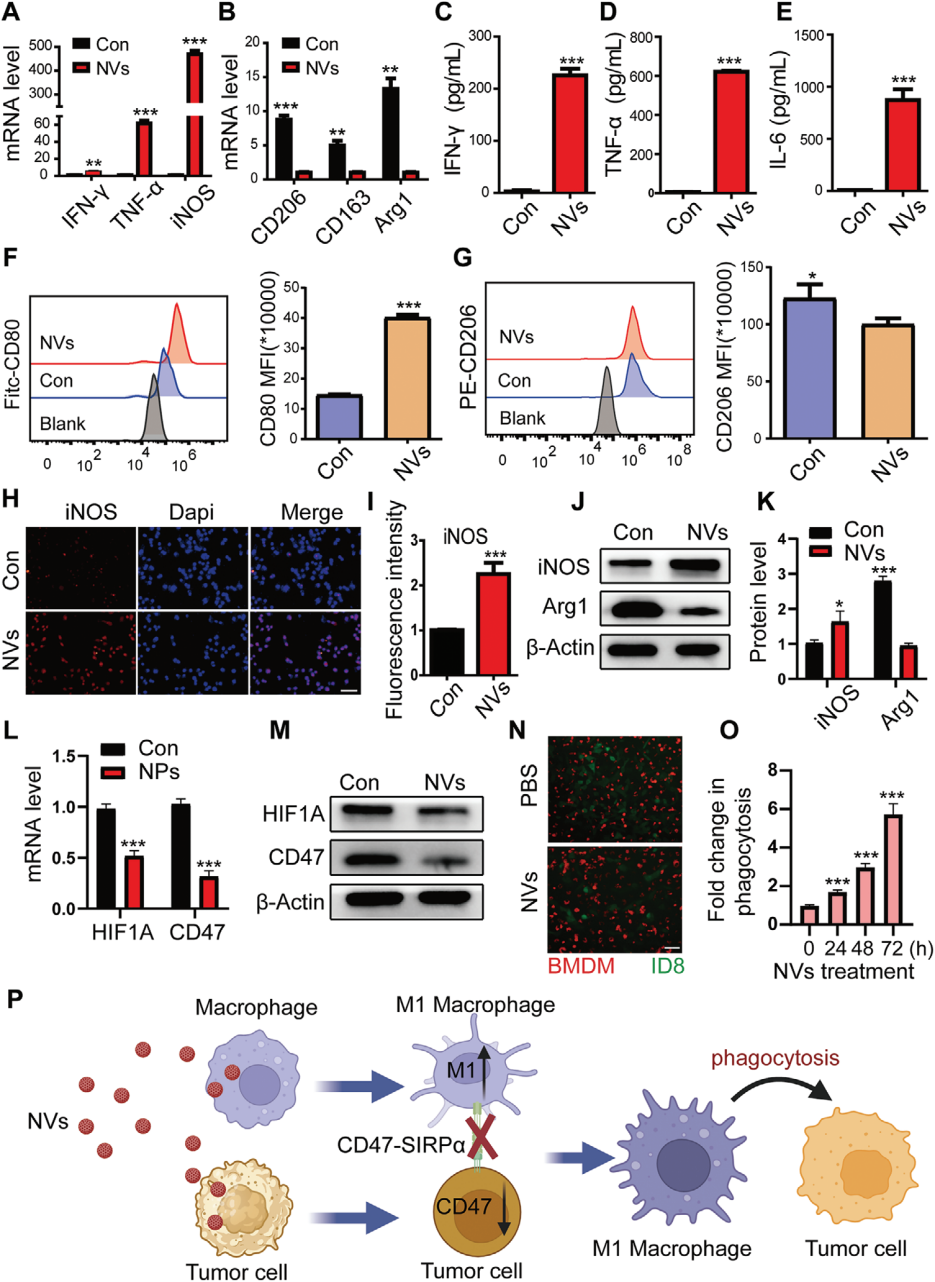
Nanovaccines induce effective antitumor immune responses. A,B) mRNA expression levels of canonical M1 and M2 macrophage biomarkers in BMDMs treated with nanovaccines for 12 h (*n* = 3). C–E) The secretion of proinflammatory cytokines IFN‐γ, TNF‐α, and IL‐6 was measured by ELISA following a 12 h treatment of BMDMs with nanovaccines (*n* = 3). F,G) Expression of CD80 and CD206 in BMDMs were assessed by flow cytometry following 12 h treatment with nanovaccines (*n* = 3). H,I) Expression of iNOS was detected by immunofluorescence following nanovaccine treatment for 12 h (*n* = 3), scale bar: 50 µm. J,K) Expression of iNOS and Arg1 was detected by western blot following nanovaccine treatment for 12 h (*n* = 3). L,M) Expression of HIF1A and CD47 was detected by RT‐PCR and western blot following nanovaccine treatment for 12 h (*n* = 3). N) Phagocytic activity of BMDMs on ID8 cells following nanovaccine treatment for 24 h, scale bar: 50 µm. O) Fold change in BMDM‐mediated phagocytosis of ID8 cells over 72 h (*n* = 3). P) Schematic representation of the synergistic antitumor effects of nanovaccines. Data are presented as mean ± SD, analyzed using an unpaired two‐sided Student's t‐test (*p < 0.05, **p < 0.01, ***p < 0.001).

Transforming TAMs into an antitumor M1‐like phenotype can alleviate the immunosuppressive TME, thereby enhancing the efficacy of antitumor immune responses.^[^ ^27^, ^28^ ^]^ To evaluate the impact of nanovaccines on macrophage polarization, we co‐incubated nanovaccines with BMDMs for 12 h. Real‐time polymerase chain reaction (RT‐PCR) analysis revealed that NVs (PLGA‐CpG@ID8‐M nanovaccines) significantly upregulated the expression of M1 macrophage markers, including interferon‐gamma (IFN‐γ), tumor necrosis factor‐alpha (TNF‐α), and inducible nitric oxide synthase (iNOS), while concurrently downregulating M2 markers such as CD206, CD163, and arginase‐1 (Arg1) (**Figure** **2**A,B). In addition, we collected supernatants to measure inflammatory cytokine levels. ELISA results demonstrated that nanovaccines markedly enhanced the production of pro‐inflammatory cytokines, including IFN‐γ, TNF‐α, and IL‐6 (Figure 2C–E). Furthermore, flow cytometry was employed to assess the expression of CD80 (an M1 marker) and CD206 in BMDMs after nanovaccine treatment. The results indicated a significant upregulation of CD80 and a corresponding downregulation of CD206, signifying a shift toward an M1‐like phenotype (Figure 2F,G). In line with the findings in BMDMs, we also examined the expression of CD86 (an M1 marker) and CD163 in RAW264.7 macrophage using flow cytometry. Similar results were observed, further supporting the immunomodulatory effects of nanovaccines on macrophage polarization (Figure S6, Supporting Information). Furthermore, the expression of iNOS and Arg1 in RAW264.7 macrophage was assessed by immunofluorescence and western blot following nanovaccine treatment. Both immunofluorescence (Figure 2H,I; Figure S7, Supporting Information) and western blot results (Figure 2J,K) showed consistent trends, further supporting the transformation of an M1‐like phenotype.

Given that nanovaccines can be internalized by tumor cells, we assessed their impact on the proliferative capacity of ID8 cells over 96 h. The CCK‐8 assay demonstrated that co‐incubation with nanovaccines did not affect tumor cell proliferation (Figure S8, Supporting Information). The CD47‐SIRPα signaling axis, a myeloid cell‐specific checkpoint for phagocytosis, is often exploited by tumor cells that upregulate CD47 to evade immune surveillance and suppress phagocytic activity. Previous studies has established that HIF1A can enhance the expression of CD47 in tumor cells.^[^ ^29^, ^30^ ^]^ In our study, NVs can downregulate CD47 and HIF1A expression in ID8 cells (Figure 2L,M). Therefore, we speculate that NVs inhibits CD47 expression by reducing HIF1A, and this finding is consistent with reports from other studies demonstrating that CpG can significantly reduce HIF1A levels in glioma tumor cells.^[^ ^31^, ^32^ ^]^ Therefore, to observe the synergistic tumor‐killing effect of nanovaccines, ID8 cells labeled with green fluorescent protein (GFP) were co‐cultured with BMDMs labeled with a cytoplasmic red fluorescent probe, and the results showed a significant reduction in tumor cells in the nanovaccine‐treated group compared to the control group (Figure 2N,O). Collectively, through a variety of assays, we demonstrate that nanovaccines achieve a synergistic antitumor effect by enhancing M1 polarization of TAMs and decreasing CD47 expression in ID8 cells (Figure 2P).

### 2.3 Nanovaccine Combined with Chemotherapy Achieves Synergistic Antitumor Effects by Remodeling TAMs

**Figure 3:**
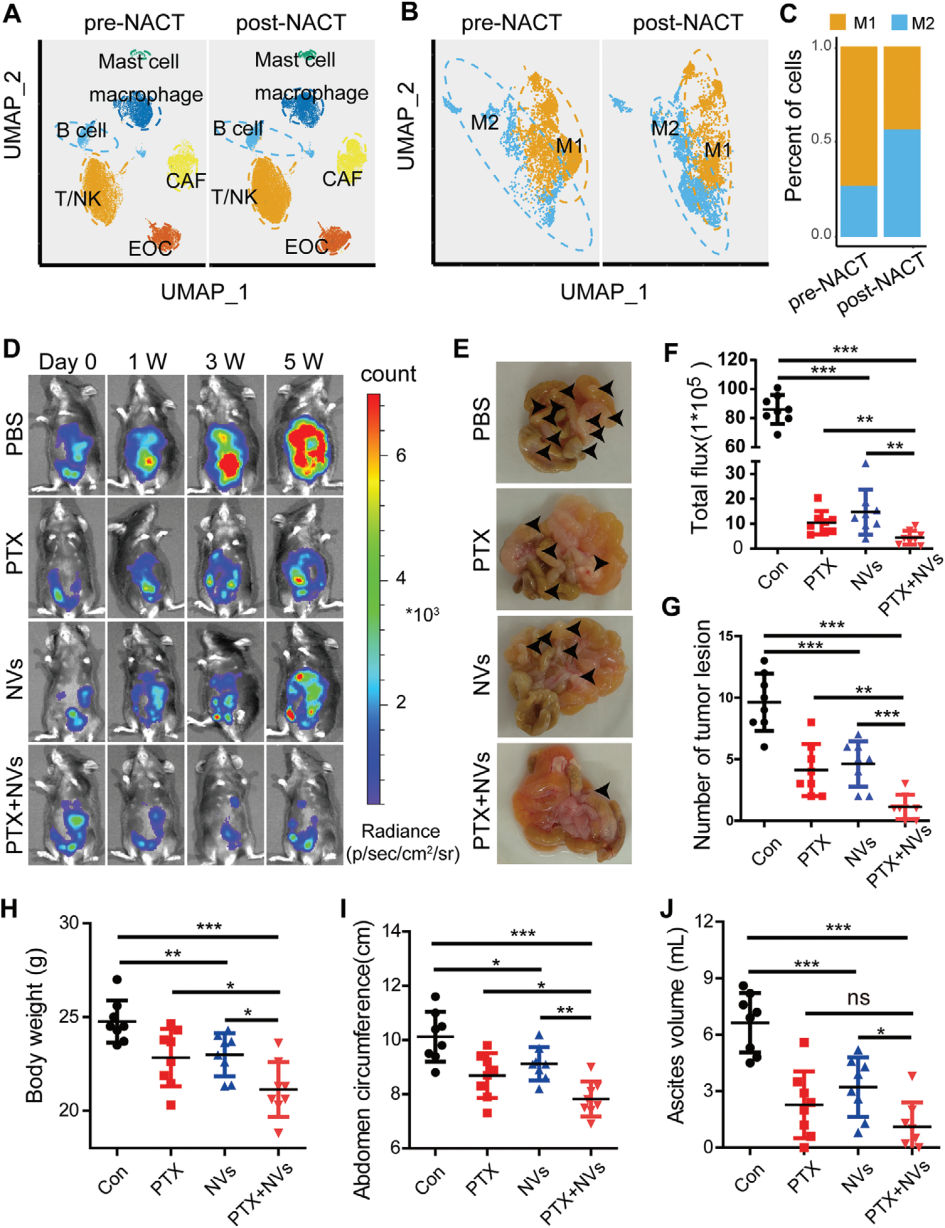
Nanovaccine combined with chemotherapy achieves synergistic antitumor effects in vivo. A) UMAP (uniform manifold approximation and projection) plot depicting all cells from prospective tumor samples of 11 patients with HGSOC before and after NACT (data from GSE165897). B,C) Classification of M1 and M2 phenotypes within the macrophage subpopulations. D,F) antitumor effects of PBS, PTX (20 mg kg^−1^, intraperitoneal injection), NVs (CpG content at 1 nmol per mouse, intravenous injection), and the combination (NVs + PTX) observed at various time points using IVIS in mouse models of ovarian cancer with intraperitoneal metastasis (*n* = 8). E,G) Assessment of the number of abdominal tumor lesions observed at the fifth week across different groups (*n* = 8). H–J) Analysis of body weight, abdominal circumference, and ascites volume at the fifth week across different groups (*n* = 8). Data are presented as mean ± SD, analyzed using an unpaired two‐sided Student's t test (ns: not significant, *p < 0.05, **p < 0.01, ***p < 0.001).

Chemotherapy remains the first‐line treatment for ovarian cancer. However, drug resistance following chemotherapy is a major factor to poor prognosis in ovarian cancer, often associated with an increase in M2‐like TAMs. To elucidate the role of TAMs in chemotherapy resistance in high‐grade serous ovarian cancer (HGSOC), we analyzed transcriptional profiles indicative of chemoresistance through single‐cell analysis of paired tumor tissue samples obtained from patients before and after neoadjuvant chemotherapy (NACT). The results demonstrated a notable increase in the proportion of M2‐like TAMs following chemotherapy treatment (**Figure** **3**A–C).

In prior in vitro experiments, we confirmed that nanovaccines can promote the transformation of TAMs into an M1‐like phenotype. Consequently, we investigated the anti‐ovarian cancer effects of nanovaccines in combination with chemotherapy in vivo. An intraperitoneal metastatic ovarian cancer model was established using ID8 cells labeled with luciferase (Luci‐ID8). After a 15 d tumor establishment period, mice were randomly assigned to four groups and treated with either PBS, PTX (paclitaxel), NVs, or a combination of NVs and PTX. The four groups of mice were treated on days D1, D5, D9, and D13, with four administrations in total. Specifically, in the (PTX + NVs) group, PTX and NVs were administered sequentially, with NVs being administered 2 d after PTX, on days D3, D7, D11, and D15. Following a 5‐week observation period, we found that both the PTX and NVs groups delayed tumor development compared to the PBS group. Notably, the (PTX+NVs) group showed a significantly greater enhancement in antitumor efficacy when compared to either the PTX or NVs group alone (Figure 3D,F). Additionally, we assessed the number of abdominal tumor lesions, and the results confirmed that NVs can reduce metastasis formation, effectively cooperating with PTX to achieve antitumor effects (Figure 3E,G). We also monitored body weight, abdominal circumference, and collected ascites from all groups, and the findings indicated that NVs were effective in mitigating weight gain associated with ascites (Figure 3H–J).

**Figure 4:**
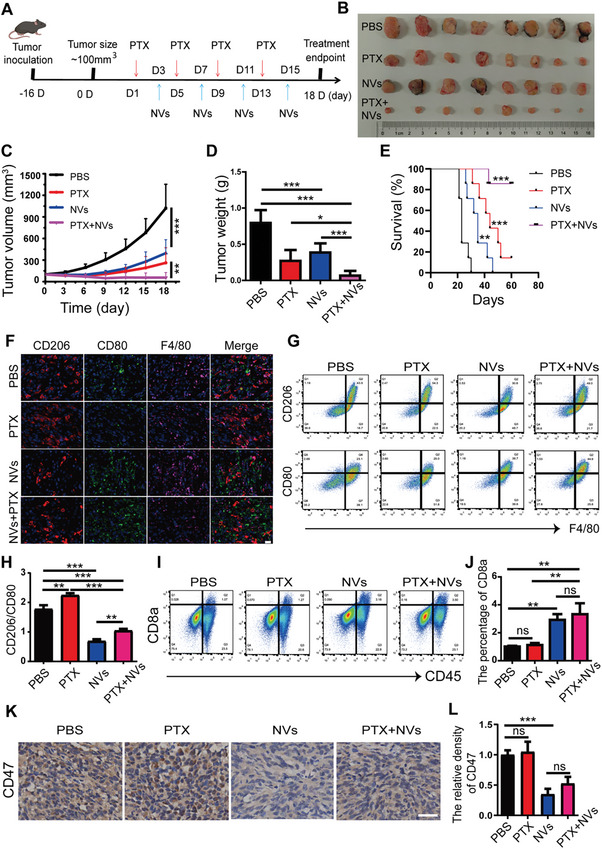
Nanovaccines induce antitumor effects in ovarian cancer by improving the immune microenvironment. A) Schematic illustration of the treatment approach for subcutaneous ovarian cancer tumor models. B–D) Changes in tumor size, volume, and weight of ID8 ovarian cancer following different treatments as indicated (*n* = 8). E) Survival curves of mice in different groups observed over a continuous 60 d period (*n* = 7). F–H) Proportions of M2/M1 macrophages (CD206/CD80) in different groups of ID8 tumor tissues, assessed by multiplex immunohistochemistry and flow cytometry (*n* = 4), scale bar: 20 µm. I,J) Proportion of CD8a^+^ T cells in different groups of ID8 tumor tissues, determined by flow cytometry (*n* = 4). K,L) Immunohistochemical staining and statistical analysis of CD47 in the different groups, scale bar: 50 µm. Data are presented as mean ± SD, analyzed using an unpaired two‐sided Student's t test (ns: not significant, *p < 0.05, **p < 0.01, ***p < 0.001).

To assess the capacity of nanovaccines to drive the polarization of TAMs towards an antitumor M1 phenotype in vivo, we established a subcutaneous tumor model of ovarian cancer (**Figure** **4**A), which was also divided into PBS, PTX, NVs, and the combination (NVs + PTX) groups. The results demonstrated that NVs effectively inhibit tumor progression (Figure 4B–D). Moreover, its synergistic application with chemotherapy exhibited pronounced antitumor efficacy, achieving an impressive survival rate of 85.7% over 60 d (Figure 4E). These findings further confirm the antitumor effects of nanovaccines, which can synergize with first‐line chemotherapy agents to overcome chemotherapy resistance, thereby providing a promising approach for ovarian cancer management.

Subsequently, we performed multiplex immunohistochemistry on ID8 tumor tissues, which further suggested that NVs effectively promoted the transformation of TAMs into the M1 phenotype (Figure 4F). Additionally, we collected tumor tissues from the different groups and digested them into single cells for flow cytometry analysis (Figure S9, Supporting Information). The flow cytometry data revealed that PTX promoted an increase in M2‐like TAMs, while NVs significantly facilitated the transformation of TAMs into the M1 phenotype, counteracting the PTX‐induced rise in M2‐like TAMs and significantly improving the TME (Figure 4G,H). As the immunosuppressive microenvironment caused by TAMs in tumor tissues is alleviated, the infiltration of CD8^+^ T cells with cytotoxic effects will increase.^[^ ^33^, ^34^ ^]^ To quantify this phenomenon, we utilized flow cytometry to assess CD8^+^ T cell infiltration in ID8 tumor tissues. The results indicated a significant increase in CD8^+^ T cell infiltration in both the NVs and (NVs + PTX) groups, compared to the PBS group (Figure 4I,J). Furthermore, we previously observed that NVs can reduce CD47 expression in ID8 cells. To further confirm this finding in vivo, we conducted immunohistochemical assays on tumor tissues following NVs treatment. Compared to the PBS group, NVs significantly reduced CD47 expression in ID8 tumor tissues (Figure 4K,L). In conclusion, nanovaccines significantly improve the tumor immune microenvironment and synergistically enhance the efficacy of chemotherapy through re‐education of TAMs.

### 2.4 Gbp2 as a Core Regulator of Macrophage M1 Polarization Induced by Nanovaccines

**Figure 5:**
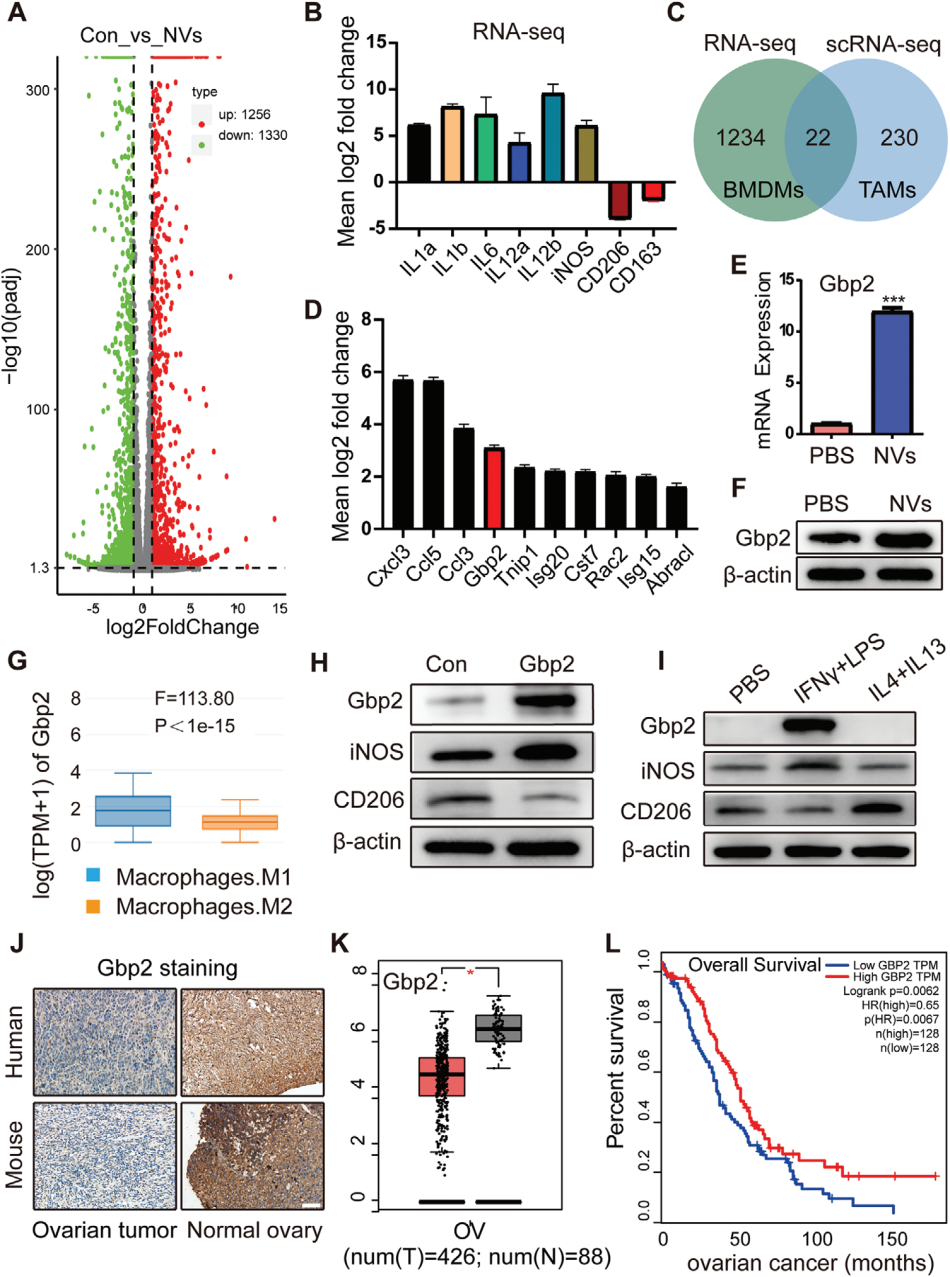
The role of nanovaccine‐induced Gbp2 in tumor prognosis. A) Number of differentially expressed genes identified in the RNA‐seq analysis. B) RNA‐seq results showing the expression levels of IL1a, IL1b, IL6, IL12a, IL12b, CD206, and CD163 in BMDMs treated with nanovaccines for 12 h. C,D) Enrichment of differentially expressed genes identified in both RNA‐seq and scRNA‐seq datasets. E,F) Detection of Gbp2 expression following BMDM activation with nanovaccines, measured by RT‐PCR and western blot. G) Expression levels of Gbp2 in M1 and M2 macrophages, obtained from the GEPIA database. H,I) Immunoblot (IB) analysis of the indicated proteins derived from RAW264.7 macrophages stably overexpressing Gbp2 (H) and those induced by (LPS + IFN‐γ) and (IL4 + IL13) (I). J) Immunohistochemical staining of Gbp2 in normal and neoplastic ovarian tissues from human and mouse samples, scale bar: 100 µm. K) Expression of Gbp2 in human ovarian cancer samples from the TCGA database. L) Prognosis of Gbp2 in ovarian cancer patients from TCGA database. Data are presented as mean ± SD, analyzed using an unpaired two‐sided Student's t test (*p < 0.05, ***p < 0.001).

Given the capacity of nanovaccines to induce macrophage M1 polarization and exert antitumor effects both in vitro and in vivo, we initiated a study to identify the key genes that regulate the M1 polarization process mediated by NVs. Firstly, we performed RNA‐seq on BMDMs treated with NVs for 12 h (**Figure** **5**A, Figure S10, Supporting Information). The RNA‐seq analysis revealed a significant upregulation of classical M1 macrophage markers, including IL1a, IL1b, IL6, IL12a, IL12b, and iNOS. Conversely, we observed a notable downregulation of CD206 and CD163, which are characteristic markers of M2 macrophages (Figure 5B). By intersecting the sets of significantly differentially expressed genes identified from this RNA‐seq data with those from the scRNA‐seq analysis mentioned in Figure 3A–C, we identified 22 genes potentially involved in promoting the transition of TAMs to the M1 phenotype (Figure 5C). Among these, we highlighted top 10 genes with significant differences. After excluding the chemokines CXCL3, CCL5 and CCL3 secreted by macrophages, Gbp2 emerged as the top target gene (Figure 5D). Gbp2, an interferon‐induced protein and a member of the guanylate‐binding protein family, plays a crucial role in regulating inflammatory signaling pathways, facilitating the immune response against bacteria, viruses, protozoa, and tumor cells.^[^ ^35^, ^36^ ^]^

To further explore the impact of nanovaccine treatment on Gbp2 expression in macrophages, we conducted additional verification using RT‐PCR and western blot analysis, confirming that the nanovaccine significantly enhances Gbp2 expression (Figure 5E,F). We also performed a cell type‐level expression analysis of Gbp2 via Gene Expression Profiling Interactive Analysis (GEPIA), which revealed that Gbp2 expression is higher in M1 macrophages compared to M2 macrophages (Figure 5G). To substantiate the role of Gbp2 in modulating M1 macrophage polarization, we utilized lentiviral infection to achieve Gbp2 overexpression in RAW264.7 macrophages. Western blot results indicated that increased Gbp2 levels were associated with upregulation of the M1 macrophage marker iNOS and downregulation of the M2 macrophage marker CD206, suggesting that Gbp2 may promote M1 polarization of macrophages (Figure 5H). To further validate this conclusion, we employed classical inducers for M1 macrophages (LPS + IFN‐γ) and M2 macrophages (IL4 + IL13) to stimulate RAW264.7 macrophages. Our findings confirmed a significant upregulation of Gbp2 in response to M1 macrophage induction (Figure 5I). Additionally, we performed immunohistochemical staining for Gbp2 on both normal and ovarian cancer tissues from human and mouse samples. The results suggested that Gbp2 is highly expressed in normal ovarian tissue, whereas it is low expressed in ovarian cancer tumor tissue (Figure 5J). This observation aligns with data from the TCGA database (Figure 5K), implying that elevated Gbp2 expression may exert an inhibitory effect on tumor progression. Importantly, increased Gbp2 levels were positively correlated with improved prognostic outcomes in ovarian cancer patients (Figure 5L). In summary, our data analysis suggests that elevated Gbp2 expression enhances the polarization of macrophages to the M1 phenotype within tumor tissues, and its high expression in ovarian cancer is associated with a more favorable prognosis.

### 2.5 Macrophages with Elevated Gbp2 Expression Significantly Inhibit Tumor Growth

**Figure 6:**
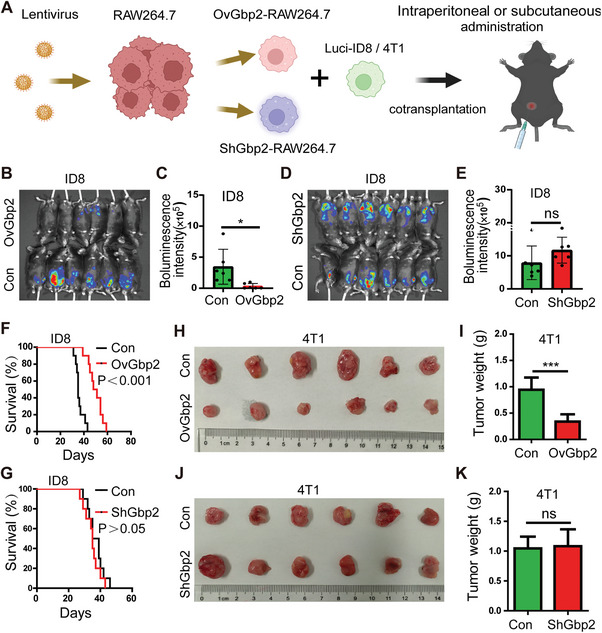
OvGbp2‐RAW264.7 macrophages inhibit the progression of solid tumor. A) Schematic representation of the construction of ID8 ovarian cancer intraperitoneal metastasis models and 4T1 breast cancer subcutaneous models. B–E) IVIS imaging results of the ID8 ovarian cancer mouse model following co‐transplantation of OvGbp2/ShGbp2‐RAW264.7 with Luci‐ID8 over a period of 15 d (*n* = 6). F,G) Survival curves of ID8 tumor‐bearing mice across different groups (*n* = 10). H–K) Tumor size measurements in the 4T1 breast cancer model following co‐transplantation of OvGbp2/ShGbp2‐RAW264.7 with 4T1 over a period of 25 d (*n* = 6). Data are presented as mean ± SD using unpaired two‐sided Student's t test (ns: not significant, *p < 0.05, ***p < 0.001).

Given that Gbp2 promotes the polarization of TAMs towards a pro‐inflammatory and tumoricidal M1 phenotype, we investigated the effect of modulating Gbp2 expression in macrophages on tumor prognosis in vivo. As illustrated in **Figure** **6**A, RAW264.7 macrophages were transduced with lentivirus to either overexpress Gbp2 (OvGbp2‐RAW264.7) or suppress Gbp2 (ShGbp2‐RAW264.7), with negative control lentivirus‐infected RAW264.7 macrophages serving as the control group. Following this, we co‐implanted these macrophages with tumor cells to assess their impact on tumor progression. When OvGbp2/ShGbp2‐RAW264.7 macrophages and Luci‐ID8 were injected intraperitoneally at a ratio of 1:4, small animal in vivo imaging was performed 2 weeks later. The results demonstrated that OvGbp2‐RAW264.7 co‐transplantation with Luci‐ID8 (OvGbp2) group significantly inhibited ovarian cancer growth compared to the control group (Con) (Figure 6B,C). In contrast, ShGbp2‐RAW264.7 co‐transplantation with Luci‐ID8 (ShGbp2) group did not show a significant difference in tumor size relative to the control group (Figure 6D,E). Furthermore, a follow‐up study revealed that OvGbp2‐RAW264.7 macrophages extended the survival of ID8 tumor‐bearing mice (Figure 6F), while no significant difference was observed in the ShGbp2 group (Figure 6G). These findings indicate that Gbp2‐induced M1 polarization of macrophages exerts an anti‐ovarian cancer effect.

To elucidate whether the Gbp2‐mediated antitumor effect is species‐specific, we also established a murine subcutaneous 4T1 breast cancer model. The experiment was divided into two cohorts: OvGbp2 group and ShGbp2 group. When OvGbp2/ShGbp2‐RAW264.7 and 4T1 were injected subcutaneously at a 1:4 ratio, the results were similar to those observed results in the ID8 ovarian cancer model (Figure 6H–K). This suggests that the Gbp2‐induced antitumor effect of macrophages may be effective across multiple tumor types.

### 2.6 Targeting Gbp2 Antagonizes the Antitumor Efficacy of Nanovaccines In Vivo

**Figure 7:**
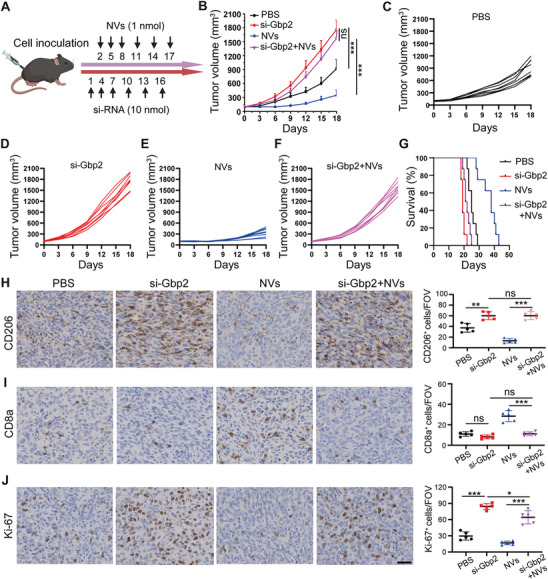
Targeting Gbp2 inhibits the antitumor efficacy of nanovaccines in vivo. A) Schematic diagram illustrating the treatment plan for mice injected subcutaneously with ID8 cells. Female C57BL/6 mice are inoculated with 7 × 10^6^ ID8 cells subcutaneously and treated with si‐Gbp2 and/or NVs for six cycles. B) Tumor volume curves of four different treatment groups (*n* = 8). C–F) Tumor volumes of mice treated with PBS, si‐Gbp2, NVs, and combined (si‐Gbp2 + NVs) therapy, measured every 3 d and plotted individually (*n* = 8). G) Kaplan–Meier survival curves showing the prognosis of different treatment groups (*n* = 8). H–J) Immunohistochemistry staining results and statistical analysis of CD206, CD8a, and Ki‐67 from different treatment groups (*n* = 5), scale bar = 20 µm. The number of CD206‐, CD8a‐, and Ki‐67‐positive cells per field of vision (FOV) in tumor tissues (200 ×) was analyzed. Data are presented as mean ± SD, analyzed using an unpaired two‐sided Student's t test (ns: not significant, *p < 0.05, **p < 0.01, ***p < 0.001).

Nanovaccines facilitate the upregulation of Gbp2, which mediates macrophage M1 polarization, thereby enhancing the antitumor efficacy in ovarian cancer in vivo. To confirm that Gbp2 plays a critical regulatory role in the anti‐ovarian cancer effects of nanovaccines, we established a subcutaneous ovarian cancer tumor model and administered Gbp2‐siRNA (si‐Gbp2) directly to the tumor sites, subsequently assessing its impact on the antitumor efficacy of NVs (**Figure** **7**A). After six interventions over 18 d, we observed that the NVs group significantly inhibited ovarian cancer progression compared to the PBS group. In contrast, the si‐Gbp2 group notably promoted tumor progression. Furthermore, the pro‐tumor effect observed in the (si‐Gbp2 + NVs) group closely resembled that of the si‐Gbp2 group, indicating that inhibition of Gbp2 counteracts the antitumor effects of NVs (Figure 7B–F). Additionally, survival analysis revealed that interference with Gbp2 significantly diminished the impact of NVs on the survival of ID8 tumor‐bearing mice (Figure 7G). Since nanovaccines can reshape TAMs, improve TME and inhibit tumor growth, we conducted experiments to collect tumor tissues 18 d post‐nanovaccines intervention. We detected the expression of CD206, Ki‐67, and CD8a using immunohistochemistry. The results showed that, compared to the PBS group, CD206‐positive TAMs and Ki‐67‐positive cells were significantly reduced in the NVs group, while the proportion of CD8a^+^ T cell were significantly increased. However, following si‐Gbp2 intervention and subsequent treatment with NVs, these antitumor effects were significantly inhibited. This resulted in an increase in CD206‐positive TAMs, a rise in Ki‐67‐positive proliferating cells, and a decline in CD8a^+^ T cell infiltration (Figure 7H–J). In conclusion, Gbp2 is a crucial regulatory factor for the antitumor effects of CpG‐based nanovaccines.

### 2.7 Nanovaccine‐Mediated Activation of the Gbp2‐Pin1‐NFkB Signaling Pathway Promotes M1 Polarization of Macrophages

**Figure 8:**
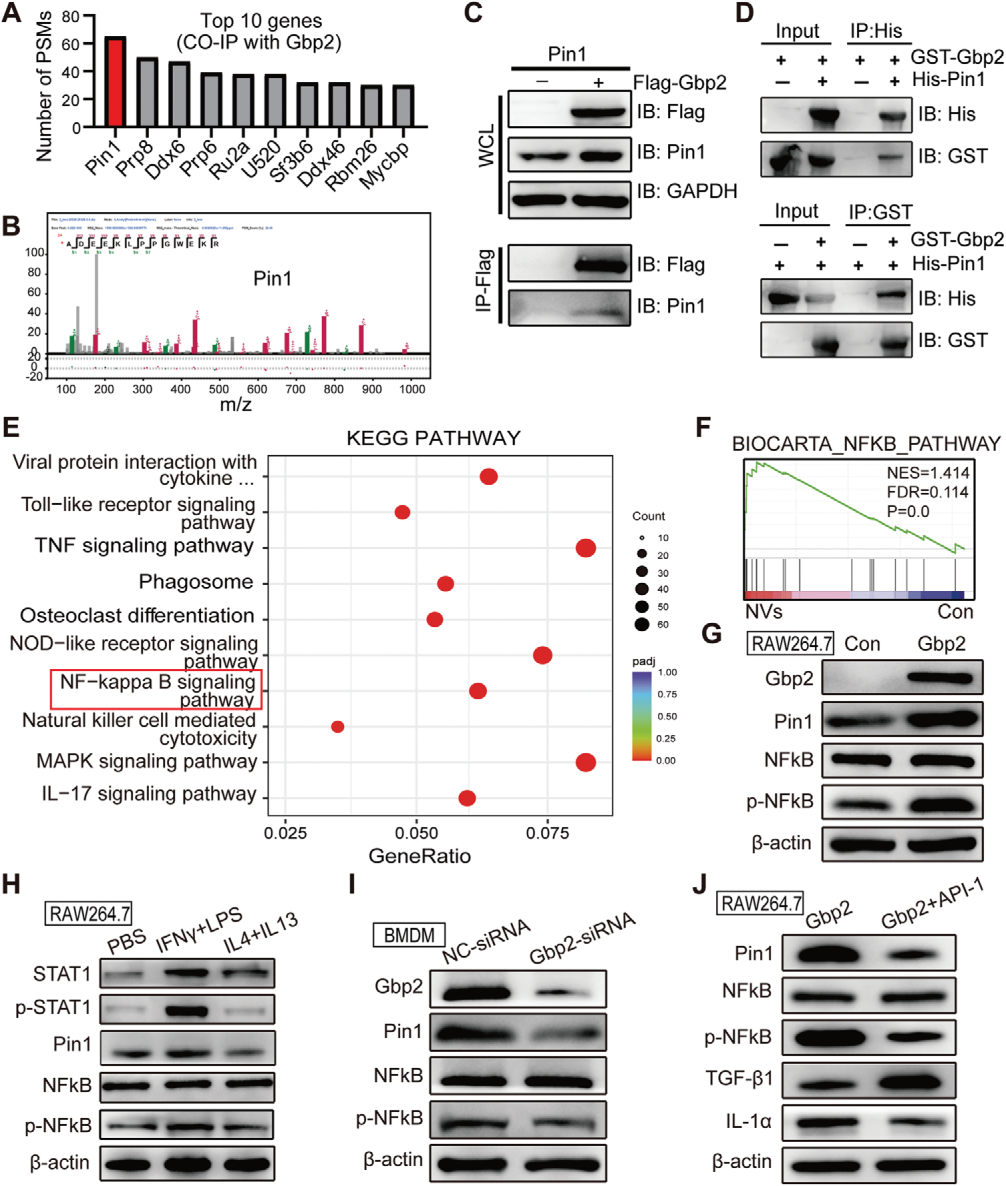
Mechanism analysis of Gbp2‐mediated M1 polarization in macrophages. A) the top 10 genes ranked by the number of peptide‐spectrum matches (PSMs) in the protein profiling analysis. B) Peptide profile of the Pin1 protein. C) IB analysis of whole‐cell lysates (WCLs) and immunoprecipitation (IP) products from RAW264.7 macrophages transfected with Flag‐Gbp2. D) In vitro binding assay using recombinant GST‐Gbp2 and His‐Pin1 proteins purified from bacteria. E) KEGG pathway analysis of RNA‐seq data. F) GSEA of the NFkB signal pathway. G) IB analysis of Gbp2, Pin1, NFkB, and p‐NFkB in RAW264.7 macrophages stably overexpressing Gbp2. H) IB analysis of STAT1, p‐STAT1, Pin1, NFkB, and p‐NFkB in RAW264.7 macrophages induced by (LPS + IFN‐γ) and (IL4 + IL13). I) IB analysis of Pin1, NFkB, and p‐NFkB in BMDMs treated with Gbp2‐siRNA. J) IB analysis of the indicated proteins following inhibition of Pin1 using API‐1 in RAW264.7 macrophages stably overexpressing Gbp2.

Through the aforementioned experiments, we have established that nanovaccines could potentially exert antitumor effects by enhancing Gbp2‐mediated M1 polarization. However, the underlying mechanism remains to be fully elucidated. Previous studies have reported that Gbp2 regulates cell function and metabolism primarily through protein interaction.^[^ ^37^, ^38^, ^39^ ^]^ To investigate the role of Gbp2 in modulating M1 macrophage polarization, we employed co‐immunoprecipitation (CO‐IP) and proteomic profiling. Our analyses identified Pin1 as the protein most abundantly interacting with Gbp2 (**Figure** **8**A,B). Pin1 typically acts as a peptidyl‐prolyl isomerase, binding to phosphorylated proteins and catalyzing the cis‐trans isomerization of proline peptide bonds, thereby influencing the structure, function, and stability of cellular proteins.^[^ ^40^ ^]^ To confirm the interaction between Gbp2 and Pin1, we induced the overexpression of Flag‐tagged Gbp2 in RAW264.7 cells. CO‐IP assays demonstrated that Gbp2 is capable of interacting with Pin1 (Figure 8C). To determine whether this interaction is direct or indirect, we expressed and purified GST‐Gbp2 and His‐Pin1 (Figure S11, Supporting Information), followed by in vitro protein interaction assays. The results indicated a direct interaction between Gbp2 and Pin1 (Figure 8D).

Next, to clarify how nanovaccines regulate M1 polarization via the Gbp2‐Pin1 pathway, we performed Kyoto encyclopedia of genes and genomes (KEGG) analysis on differentially expressed genes following nanovaccine treatment. Our analysis revealed a significant enrichment of the NFκB signaling pathway, known to promote macrophage M1 polarization (Figure 8E). Gene set enrichment analysis (GSEA) further indicated a marked enrichment of proteins within the NFκB signaling pathway in the NVs‐treated group (Figure 8F). Previous studies have reported that Pin1 can activate the NFkB signaling pathway through its cis‐trans isomerase activity.^[^ ^41^, ^42^ ^]^ Therefore, we speculate that nanovaccines may activate macrophages to the M1 phenotype by regulating the Gbp2‐Pin1‐NFkB signaling pathway.

To test this hypothesis, we overexpressed Gbp2 in RAW264.7 macrophages. The results showed that OvGbp2‐RAW264.7 led to significant increases in the expression of both Pin1 and phosphorylated NFκB (p‐NFκB) (Figure 8G). In addition, during the polarization of macrophages toward the M1 phenotype, both Pin1 and p‐NFκB expressions were notably elevated (Figure 8H). In contrast, siRNA‐mediated knockdown of Gbp2 in BMDMs resulted in reduced expression of both Pin1 and p‐NFkB (Figure 8I). Furthermore, the addition of the Pin1 inhibitor (API‐1) to OvGbp2‐RAW264.7 significantly counteracted the upregulation of Pin1 and p‐NFkB (Figure 8J). In total, our findings demonstrate that nanovaccines promote Gbp2 upregulation and facilitate the recruitment of Pin1, thereby activating the NFκB signaling pathway and enhancing M1 polarization of macrophages.

## 3 Conclusion

In this study, the CpG‐based nanovaccines we have developed possesses tumor‐targeting capabilities that enable it to reprogram TAMs into the M1 phenotype, known for its antitumor properties. This transformation mitigates the immunosuppressive effects of the TME and subsequently prolongs the survival of tumor‐bearing mice. While chemotherapy remains the first‐line treatment for ovarian cancer and demonstrates reasonable efficacy, it is associated with a high recurrence rate of 70–80%.^[^ ^43^ ^]^ Our study reveals that chemotherapy can significantly increase the population of M2‐like TAMs, thereby fostering an immunosuppressive microenvironment. In contrast, nanovaccines can counteract this chemotherapy‐induced immunosuppression by reducing M2‐like TAMs and enhancing macrophage phagocytosis through the downregulation of CD47 expression in ID8 tumor cells. Therefore, the combination of chemotherapy and nanovaccines holds promise as a potential strategy for ovarian cancer treatment. Additionally, we explored the specific mechanisms by which nanovaccines reshape TAMs. Using multi‐omics sequencing alongside various tumor‐bearing mouse models, we found that nanovaccines promote the M1 polarization of macrophages via the Gbp2‐Pin1‐NF‐κB signaling cascade, thereby achieving antitumor effects.

In conclusion, we provide a systematic elucidation of a novel mechanism through which nanovaccines modulate TAMs for the first time. This discovery provides a new direction for ovarian cancer immunotherapy, and the combination of nanovaccines with chemotherapy presents a novel therapeutic strategy for ovarian cancer, deserving further exploration and validation in future studies. In addition, some limitations were identified in this study. For instance, we noted the lack of systematic studies on the effects of nanovaccines on other cell types in the TME, such as DCs, natural killer cells, and B cells. Future research should include more comprehensive, multi‐dimensional analyses. Additionally, the long‐term effects and potential drug resistance of nanovaccines may not have been fully explored in this study. Future work should focus on examining the effects of long‐term use and investigating the mechanisms of drug resistance. Finally, regarding clinical translation, the conclusions of this study will require further validation through extensive preclinical studies and clinical trials.

## 4 Experimental Section

### Material

PLGA (Mw 7000−17000, lactide/glycolide 50:50) and dichloromethane were purchased from Aladdin Biochemical Technology Co., LTD. ICG (Indocyanine green) and LPS (Lipopolysaccharides, L4391) were purchased from Sigma‐Aldrich. CpG 1826 (5′‐tccatgacgttcctgacgtt‐3′, all oligodeoxynucleotides modified by thiophosphate) and all mRNA primers were synthesized by Shenggong Bioengineering (Shanghai) Co., Ltd. The Cell Counting Kit‐8 was purchased from Dojindo Laboratories. CellTrace Red CMTPX was purchased from Yeasen Biotechnology (Shanghai) Co., Ltd. ELISA kits of IL‐6, TNF‐α, and IFN‐γ were purchased from MULTI SCOENCES. Paclitaxel (A4393) was purchased from APExBIO. API‐1(680622‐70‐2) and anti‐Flag magnetic bead (HY‐K0207) were purchased from MCE (MedChemExpress). Cytokines IL4, IL13, and IFNγ were purchased from peprotech. The GST‐tag Protein Purification Kit, His‐tag Protein Purification Kit, IPTG(isopropyl‐β‐D ‐thiogalactoside), polybrene, cell lysis buffer for Western and IP, Membrane and Cytosol Protein Extraction Kit, DiI, and BCA Protein Assay Kit were purchased from Beyotime Biotechnology. Zombie NIRTM Fixable Viability Kit (423105), anti‐mouse CD16/32 (101320), PerCP/Cyanine5.5 anti‐mouse CD45 (157208), APC anti‐mouse/human CD11b (101211), PE/Cyanine7 anti‐mouse F4/80 (123114), PE anti‐mouse CD206 (141706), Brilliant Violet 421 anti‐mouse CD80 (104726), Alexa Fluor 488 anti‐mouse CD8a Antibody (100726), PE anti‐mouse CD163 Antibody (156703), FITC anti‐mouse CD80 Antibody (104705), and APC anti‐mouse CD86 Antibody (105011) for flow cytometry were purchased from BioLegend. CD206 (ab64693) and F4/80 (ab100790) were purchased from abcam. CD80 (66406‐1‐Ig), Arg1(16001‐1‐AP), iNOS (18985‐1‐AP), β‐Actin (66009‐1‐Ig), Gbp2 (27299‐1‐AP), FLAG (20543‐1‐AP), CD206 (18704‐1‐AP), Pin1(10495‐1‐AP), TGFβ1(21898‐1‐AP), GAPDH (60004‐1‐Ig), GST (66001‐2‐Ig), CD47 (20305‐1‐AP), and His (66005‐1‐Ig) were purchased from Proteintech. NFkB (8242), p‐NFkB (3033), and HIF1A (36169) were purchased from CST (Cell Signaling Technology). STAT1 (A12075), p‐STAT1 (AP0054), and IL1a (A2170) were purchased from ABclonal. CD8 (GB15068) and Ki‐67 (GB111499) were purchased from Servicebio.

### Cell and Cell Culture

The ID8 cell line was provided by K. Roby (Department of Anatomy and Cell Biology, University of Kansas). The RAW264.7, 4T1, 3T3‐L1, and HeLa cell lines were purchased from American Tissue Culture Collection (ATCC). ID8, RAW264.7, 3T3‐L1, and HeLa cell lines were cultured in Dulbecco's modified Eagle medium (DMEM) (Gibco) supplemented with 10% fetal bovine serum (FBS). 4T1 cell line were cultured in RPMI‐1640 (Gibco) supplemented with 10% FBS. BMDMs isolated from femurs and tibias of female C57BL/6 mice (8 weeks) were cultured in DMEM medium supplemented with 10% FBS and 20 ng mL^−1^ M‐CSF. All the media were supplemented with 100 U mL^−1^ penicillin and 100 µg mL^−1^ streptomycin, and all cells were cultured in a 37 °C incubator with 5% CO_2_.

### Animal Experiment

All animal experimental operations were conducted according to the Guide for the Care and Use of Laboratory Animals of Tongji Hospital (TJH‐202207047). Female C57BL/6 mice (6−8 weeks) and Balb/c mice (6−8 weeks) were purchased from SPF (Beijing) biotechnology Co. Ltd and were housed under specific pathogen‐free conditions. Tumor tissues from patients were obtained from Tongji Hospital, and the sample collection was approved by the local ethics committee (TJ‐IRB20210319) and all participants provided informed consent. For the ovarian cancer model of intraperitoneal metastasis depicted in Figure 3, the model was established using 1 × 10^7^ Luci‐ID8 cells. The subcutaneous ovarian cancer tumor models shown in Figures 1, 4, and 7 were established using 7 × 10^6^ ID8 cells. Luci‐ID8 ovarian cancer intraperitoneal metastasis models and 4T1 breast cancer subcutaneous models cotransplantation with RAW264.7 cell for Figure 6 were established using 8 × 10^6^ ID8 cells and 1 × 10^6^ 4T1 cells, respectively. Tumor size, body weight, and general health conditions were monitored according to previously published article,^[^ ^44^, ^45^ ^]^ and tumor volume (mm^3^) was calculated by the formula: volume (mm^3^) = 0.5 × (width)^2^ × length.

### Preparation of PLGA‐CpG@ID8‐M Nanovaccines

The synthesis of the nanovaccines was performed following the ultrasound double emulsion method described in the previous work.^[^ ^17^ ^]^ Briefly, 134 µg of CpG was added to 50 µL of double‐distilled water (ddH₂O), and 10 mg of PLGA dissolved in 0.5 mL of dichloromethane was added to the 50 µL CpG solution. This mixture was sonicated at 20% power (Sonics & Materials, Inc.) for eight cycles on ice, resulting in a total volume of 0.55 mL of a milky white suspension (*T*on = 3 s, *T*off = 10 s). Subsequently, 8 mL of ddH₂O was added to this suspension, and sonication was continued at 35% power for 15 cycles on ice (*T*on = 3 s, *T*off = 10 s). The mixture was then magnetically stirred at 600 rpm overnight to facilitate the evaporation of the organic solvent. The PLGA‐CpG nanoparticles were collected by centrifugation at 5000 rpm for 10 min using ultrafiltration tubes with a molecular weight cut‐off of 30 kD and washed three times with ddH₂O to remove any unbound CpG.

The ID8‐M was obtained according to the previously published methods by using Membrane and Cytosol Protein Extraction Kit according to the manufacturer's instructions.^[^ ^46^ ^]^ The membrane protein concentration of ID8‐M was determined using a BCA protein assay kit. The resulting ID8‐M was resuspended in PBS or ddH₂O and stored at 4 °C or ‐80 °C for later use. To coat the PLGA‐CpG nanoparticles with ID8‐M, a solution of ID8‐M (0.2 mL, 5 mg mL^−1^) was co‐extruded through polycarbonate membranes of 800 nm, 400 nm, and 200 nm in size with the PLGA‐CpG nanoparticles (0.8 mL, 2.5 mg mL^−1^) for 8–10 passes. The resulting PLGA‐CpG@ID8‐M nanovaccines were collected by centrifugation at 10 000 *g* for 10 min and resuspended in PBS or ddH₂O for further use.

### Cytotoxicity Assessment of PLGA‐CpG@ID8‐M Nanovaccines In Vivo and In Vitro

RAW264.7 macrophages were seeded at a density of 5 × 10^3^ cells per well in 96‐well plates. Once the cells reached approximately 70% confluence, they were treated with a range of CpG nanovaccine concentrations (0, 0.1, 0.25, 0.5, 1.0, 2.0, 4.0, and 8.0 µm). After a 24 h incubation period, the supernatant was removed and replaced with 100 µL of fresh culture medium containing 10 µL of CCK‐8 reagent. The cells were then incubated for an additional 2 h at 37 °C in the dark. Absorbance was measured at 450 nm using a microplate reader (BioTek, ELx808). To evaluate the cytotoxicity of nanovaccines on other cell types, BMDMs, ID8, 4T1, HeLa, and 3T3 cells were exposed to a CpG concentration of 0.5 µm under similar incubation conditions. For in vivo safety evaluation, the nanovaccines (1 nmol of CpG per mouse) were administered intravenously to female C57BL/6 mice (8 weeks old). Biochemical assays and pathological examinations were conducted on the heart, liver, spleen, lungs, and kidneys.

### Tumor Targeting of PLGA‐CpG@ID8‐M Nanovaccines In Vivo

ID8 cells (7 × 10^6^) were subcutaneously implanted into the right flank region of 8 week old female C57BL/6 mice. Upon reaching a tumor volume of approximately 100 mm^3^, the mice were administered nanovaccines containing ICG at a dosage of 1.0 mg kg^−1^ via intravenous injection. Six hours post‐injection, the near‐infrared fluorescence of the ID8 tumor was visualized using an IVIS imaging system. Furthermore, to indicate the percentage of nanovaccine reaching tumor sites, tumor tissue was dissected, weighed and homogenized in DMSO solution to extract ICG, and the concentrations of ICG were determined by fluorometry and presented as percentage injected dose per gram of tissue (ID g^−1^).

### Effect of Nanovaccines on Macrophage M1 Polarization In Vitro Detected by mRNA and ELISA

0.5 µm nanovaccine was added to the BMDMs cultured in six‐well plates, and total RNA was extracted after co‐incubation for 12 h. RT‐PCR was performed using the CFX Connectsystem (Bio‐Rad). The primer sequences for IFNγ, TNFα, iNOS, CD206, CD163, Arg1, and Gapdh are listed in Table S1 (Supporting Information). In addition, the concentrations of IL‐6, TNF‐α, and IFN‐γ in the supernatants were measured according to the manufacturer's instructions for the ELISA kits.

### Immunofluorescence Assay

RAW264.7 macrophages were seeded in 12‐well plates and treated with nanovaccines for 12 h. The cells were then fixed with 4% paraformaldehyde for 15 min and permeabilized using 0.1% Triton X‐100 for 20 min. After permeabilization, the cells were incubated overnight at 4 °C with primary antibodies diluted in PBS containing 5% bovine serum albumin (BSA). Following washing, CY3‐conjugated secondary antibodies were applied, and immunofluorescence images were captured using a fluorescence microscope.

### Immunoblot and Immunoprecipitation Analyses

Cell lysates were prepared using a RIPA lysis buffer supplemented with proteinase and phosphatase inhibitors. The total protein content of the whole‐cell lysates was quantified using a BCA assay kit. A total of 25 µg of protein was separated via SDS‐polyacrylamide gel electrophoresis (SDS‐PAGE) on 10% acrylamide gels, then transferred onto PVDF membranes (Millipore) utilizing the Trans‐Blot Turbo transfer system (Bio‐Rad). Immunoblotting was performed with the indicated antibodies at concentrations recommended by the manufacturer. Protein bands were detected using an enhanced chemiluminescence reagent (Thermo Scientific).

For immunoprecipitation (IP), cells were lysed in IP lysis buffer containing proteinase and phosphatase inhibitors on ice for 20–30 min. The lysates were centrifuged at 12000 rpm at 4 °C for 10 min. The supernatant was collected and incubated overnight at 4 °C with Anti‐Flag magnetic beads. The immunocomplexes were washed five times with 1% TBST. Proteins immobilized on the beads were eluted with 2 × loading buffer by heating at 100 °C for 10 min and then analyzed by western blot.

### Flow Cytometry Analysis of Tumor Tissues

Tumor tissue samples were carefully dissected into small fragments using ophthalmic scissors. The fragments were then immersed in DMEM supplemented with 1.0 mg mL^−1^ Collagenase IV and 50 U mL^−1^ DNase I, and incubated on a shaker set at 120 rpm for 2 h at 37 °C. The resulting cell pellets and debris were collected by centrifugation at 1200 rpm for 5 min. After washing with PBS, the remaining cells and minor debris were digested with 0.25% trypsin for 8 min. Digestion was terminated with complete medium, followed by another centrifugation at 1200 rpm for 5 min. Finally, the cells were filtered through 70 µm nylon strainers to prepare single‐cell suspensions. After treatment with a viability dye and a blocking antibody, the cells were analyzed using flow cytometry with antibodies at the manufacturer‐recommended concentrations. M1 macrophages were defined as CD45^+^CD11b^+^F4/80^+^CD80^+^, M2 macrophages as CD45^+^CD11b^+^F4/80^+^CD206^+^, and CD8a^+^ T cells as CD45^+^ CD8a^+^.

### Multiplex Immunohistochemistry

Paraffin‐embedded sections of the ovarian tumor were subjected to heat treatment at 60 °C for 1 h. The sections were deparaffinized using xylene and rehydrated through a series of graded ethanol solutions (100%, 95%, 80%, and 75%), followed by rinsing with distilled water. After antigen retrieval, the slides were treated with 3% hydrogen peroxide to quench endogenous peroxidase activity and subsequently blocked with a 5% BSA solution to prevent non‐specific binding. Primary antibodies specific for CD206, CD80, and F4/80 were applied, followed by HRP‐conjugated secondary antibodies and sequential incubation with fluorescent tyramine substrates CY3, 488, and CY5. The nuclei were counterstained with DAPI to visualize cellular morphology, and the slides were sealed with an anti‐fluorescence quenching agent to maintain fluorescence integrity. Stained tumor tissue images were observed under a microscope.

### Statistical Analysis

Statistical analyses were performed using GraphPad Prism 8 (GraphPad Software). Data are presented as mean ± standard deviation (SD). Results were analyzed using an unpaired two‐sided Student's t test, while Kaplan–Meier analysis and log‐rank test were conducted to analyze the animal survival benefit. A value of *P* < 0.05 are considered significant (ns: no significance, *P < 0.05, **P < 0.01, ***P < 0.001).

## Conflict of Interest

The authors declare no conflict of interest.

## Author Contributions

J.X., J.H., H.X., and Q.W. contributed equally to this work. J.X. was responsible for data acquisition, analysis, investigation, and writing original draft. J.H., H.X., and Q.W. were responsible for data acquisition and analysis; J.Z., Y.C., G.F., H.G., Z.H., S.W., R.X., W.O., and S.W. provided the methods, resources, and advice for the study. L.Z., P.X., W.Z., and M.W. were responsible for the supervision, conceptualization, and design of the study.

## Supporting information



Supporting Information

## Data Availability

The data that support the findings of this study are available from the corresponding author upon reasonable request.
