# Supplementary material for: CpG‐Based Nanovaccines Enhance Ovarian Cancer Immune Response by Gbp2‐Mediated Remodeling of Tumor‐Associated Macrophages
Source: Adv Sci (Weinh). 2025 Feb 22;12(15):2412881. doi: 10.1002/advs.202412881 (PMC12005807; doi:10.1002/advs.202412881)
Supplement: Supplementary file 1 — Supporting Information [file ADVS-12-2412881-s001.docx]

**Supporting Information**

CpG-based Nanovaccines Enhance Ovarian Cancer Immune Response by Gbp2-mediated Remodeling of Tumor-Associated Macrophages

Jiaqiang Xiong^1#^, Juyuan Huang^1#^, Hanxiao Xu^2#^, Qiuji Wu^3#^, Jiahui Zhao^1^, Yurou Chen^1^, Guanlan Fan^1^, Haotong Guan^1^, Rourou Xiao^1^, Zhaojin He^4^, Siqi Wu^4^, Wenliang Ouyang^4^, Shixuan Wang^5^, Lu Zhang^6*^, Peng Xia^7*^, Wei Zhang^1*^, Meng Wu^5*^

1. Department of Obstetrics and Gynecology, Zhongnan Hospital of Wuhan University, Wuhan 430071, China

2. Department of Gastrointestinal Oncology, Zhongnan Hospital of Wuhan University, Wuhan 430071, China

3. Department of Radiation and Medical Oncology, Hubei Key Laboratory of Tumor Biological Behavior, Hubei Provincial Clinical Research Center for Cancer, Zhongnan Hospital of Wuhan University, Wuhan, 430071, China

4. The Second Clinical College of Wuhan University, Wuhan, 430071, China

5. Department of Obstetrics and Gynecology, Tongji Hospital, Tongji Medical College, Huazhong University of Science and Technology, Wuhan 430032, China

6. Hubei Key Laboratory of Radiation Chemistry and Functional Materials, School of Nuclear Technology and Chemistry & Biology, Hubei University of Science and Technology, Xianning 437100, China

7. Department of Hepatobiliary & Pancreatic Surgery, Zhongnan Hospital of Wuhan University, Wuhan 430071, China

#contributed equally to this work

*Corresponding author: Lu Zhang, email: luzhang@hbust.edu.cn; Peng Xia, email: drxiapeng@whu.edu.cn; Wei Zhang, email: zhangwei2199@znhospital.cn; Meng Wu, email: mengwu@tjh.tjmu.edu.cn

**
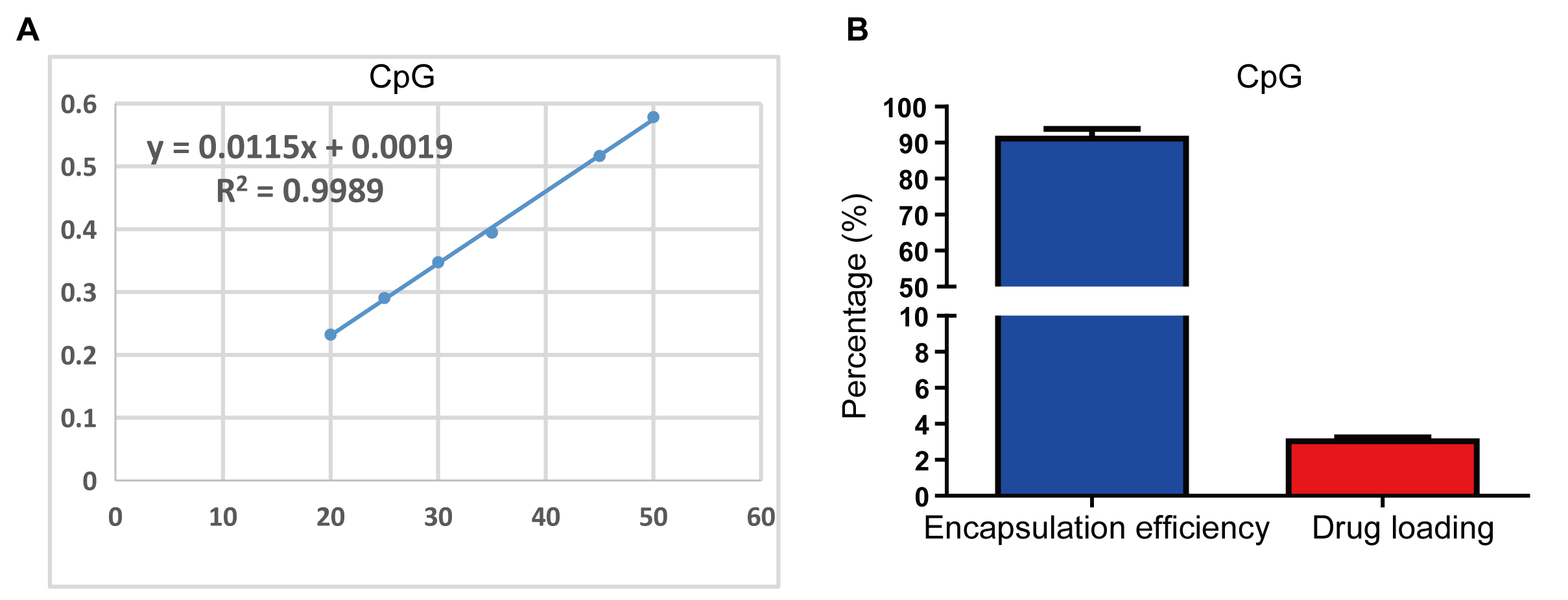
**

**Figure S1.** Loading efficiency of CpG within PLGA using the ultrasound double emulsion technique. A) Standard curve of CpG detected by a UV–vis spectrophotometer. B) Loading efficiency and loading capacity of CpG in PLGA (n = 3).


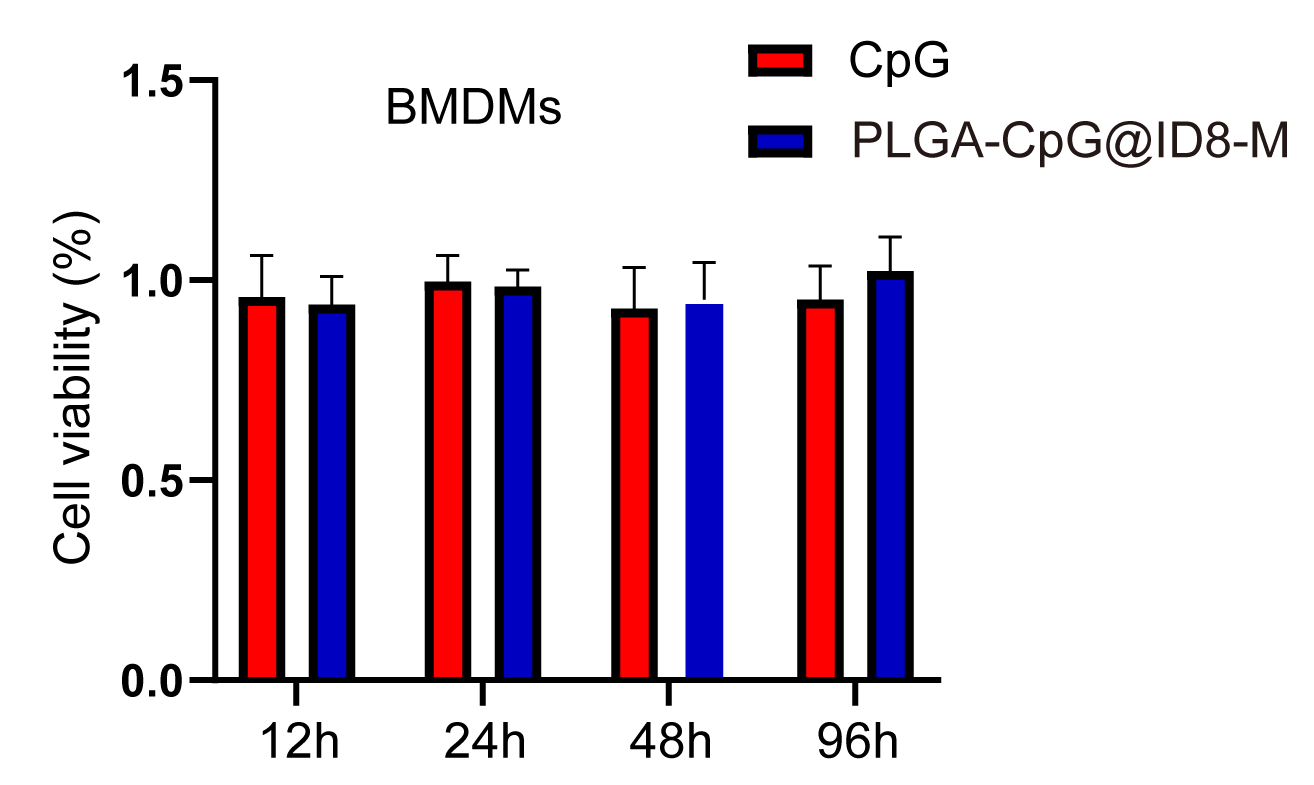


**Figure S2.** Cytotoxicity assessment of CpG and PLGA-CpG@ID8-M nanovaccine on BMDMs over a 96-hour period (n = 4).

**
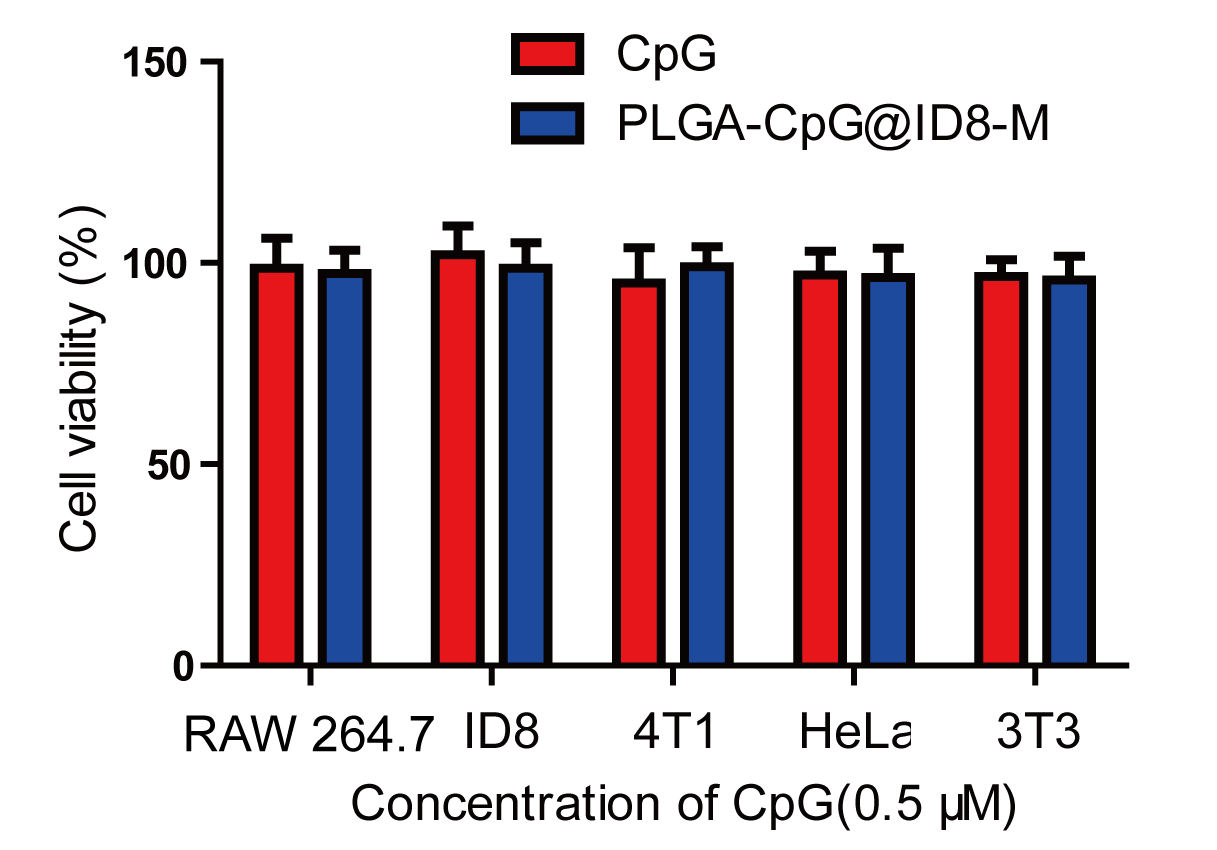
**

**Figure S3.** Cytotoxicity assessment of CpG and PLGA-CpG@ID8-M nanovaccine on RAW264.7, ID8, 4T1, HeLa, and 3T3 cells in vitro for 24 hours (n = 4).


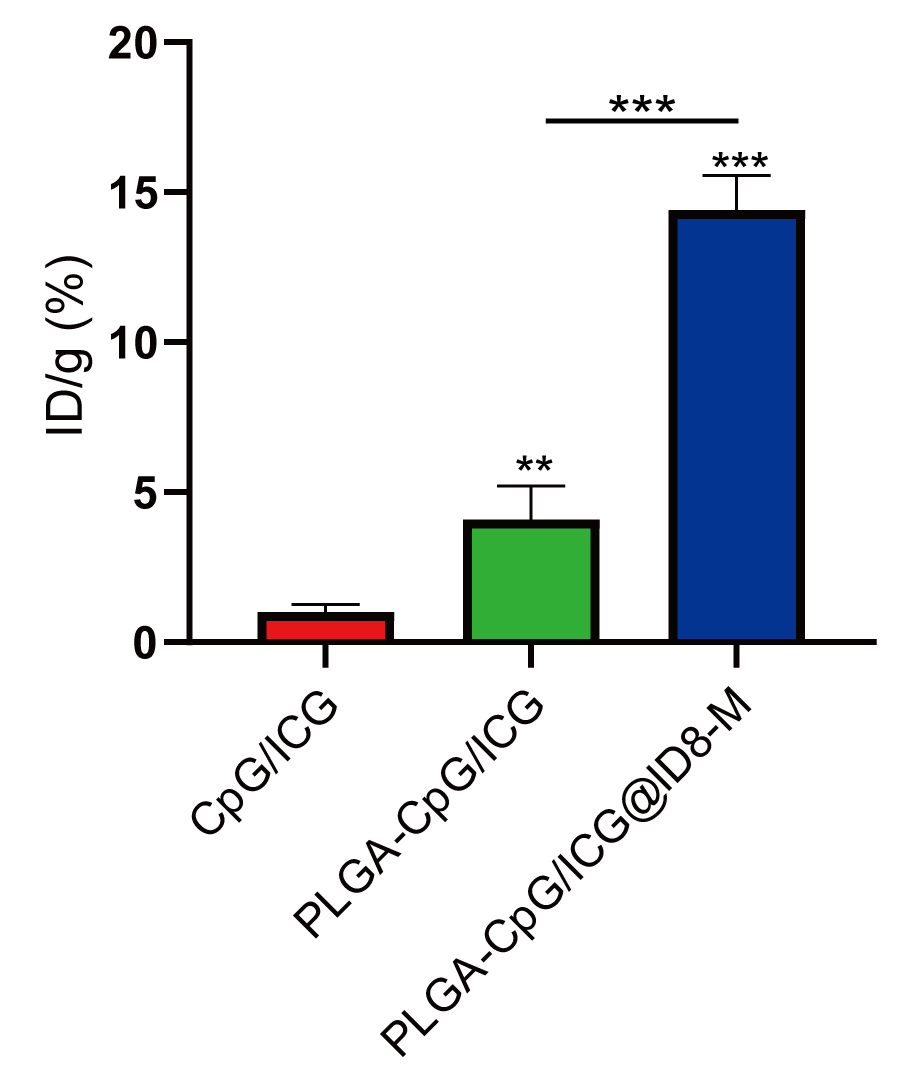


**Figure S4.** The percentage of nanovaccine aggregation in the tumor 6 hours after intravenous administration (n = 3). Data are presented as mean ± SD, analyzed using an unpaired two-sided Student’s t test (***p* < 0.01, ****p* < 0.001).

**
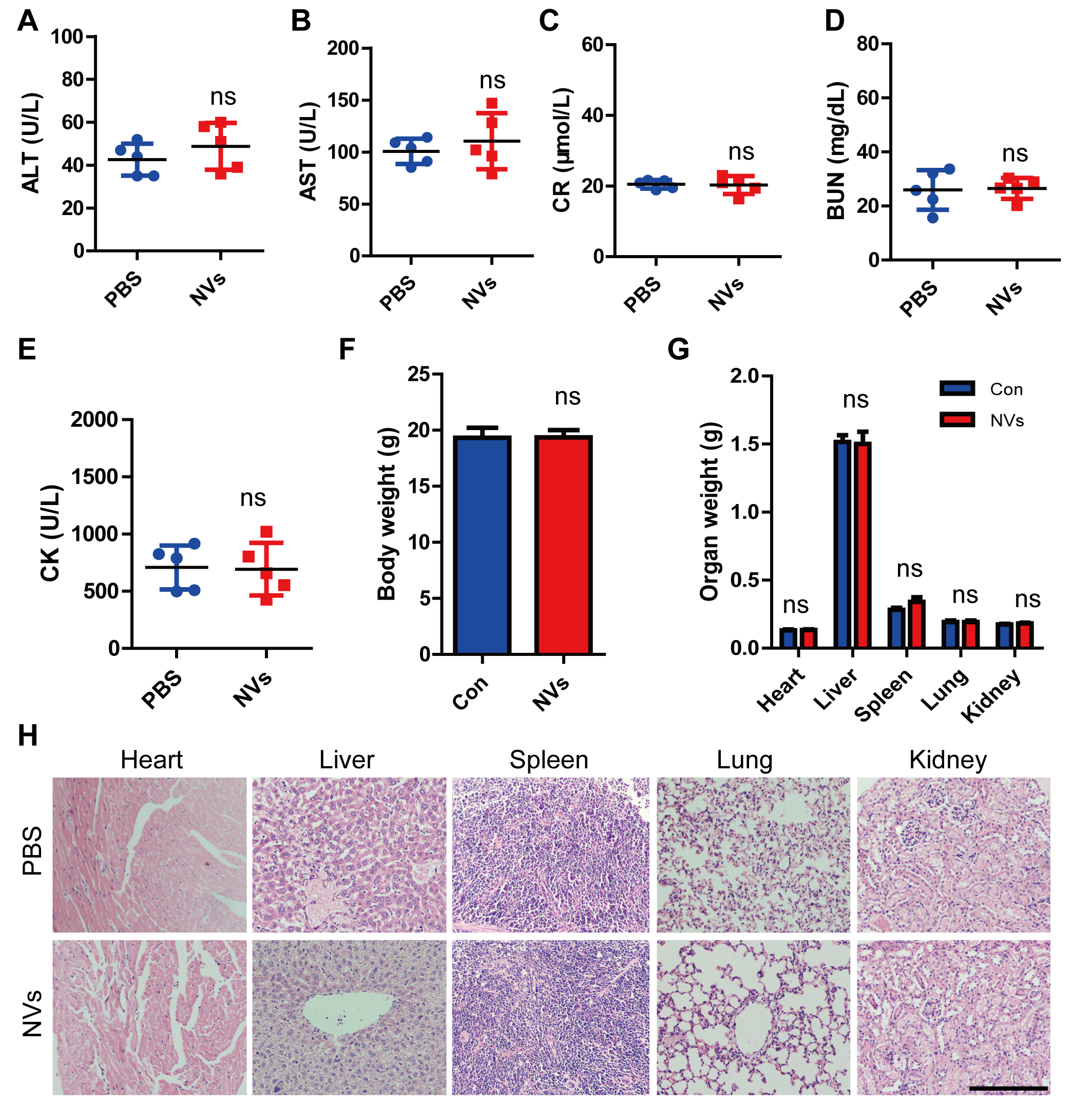
**

**Figure S5.** Biosafety evaluation of the nanovaccines in vivo. A-E) Biochemical index analysis of liver function (ALT, AST), kidney function (CR, BUN), and cardiac function (CK) (n = 5). F) Body weight of female C57BL/6 mice after treatment with the nanovaccine (CpG content at 1 nmol per mouse, intravenously injected) administered three times on days D1, D5, and D9 (n = 5). G, H) Organ weight and histological examination of the heart, liver, spleen, lungs, and kidneys after nanovaccine treatment (n = 5), scale bar: 200 μm. Data are presented as mean ± SD, analyzed using an unpaired two-sided Student’s t test (ns: not significant).

**
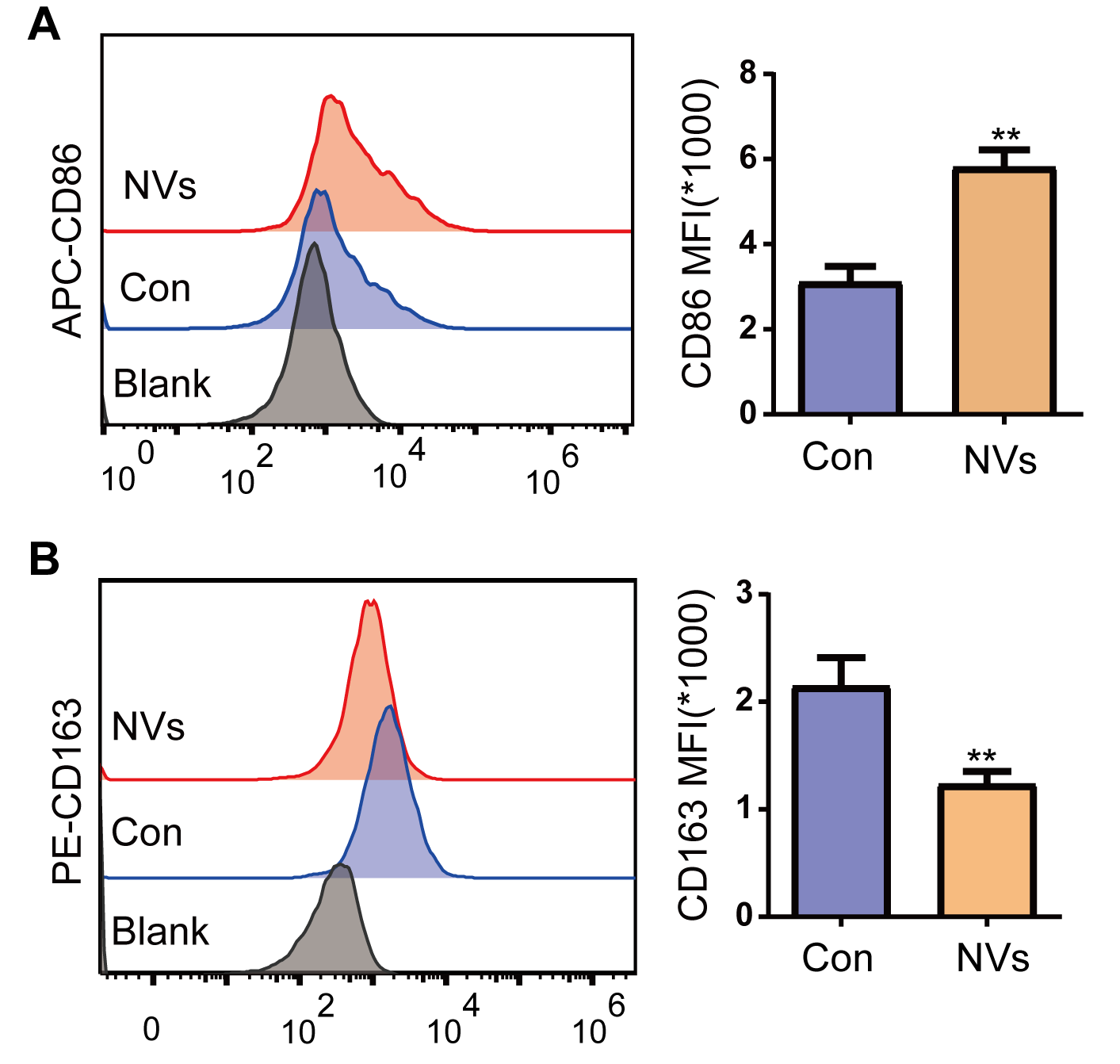
**

**Figure S6.** Detection of RAW264.7 macrophage polarization treated with the nanovaccines using flow cytometry. A, B) Flow cytometry analysis of CD86 and CD163 expression in RAW264.7 macrophages following 12-hour treatment with the nanovaccines (n = 3). Data are presented as mean ± SD, analyzed using an unpaired two-sided Student’s t test (***p* < 0.01).

**
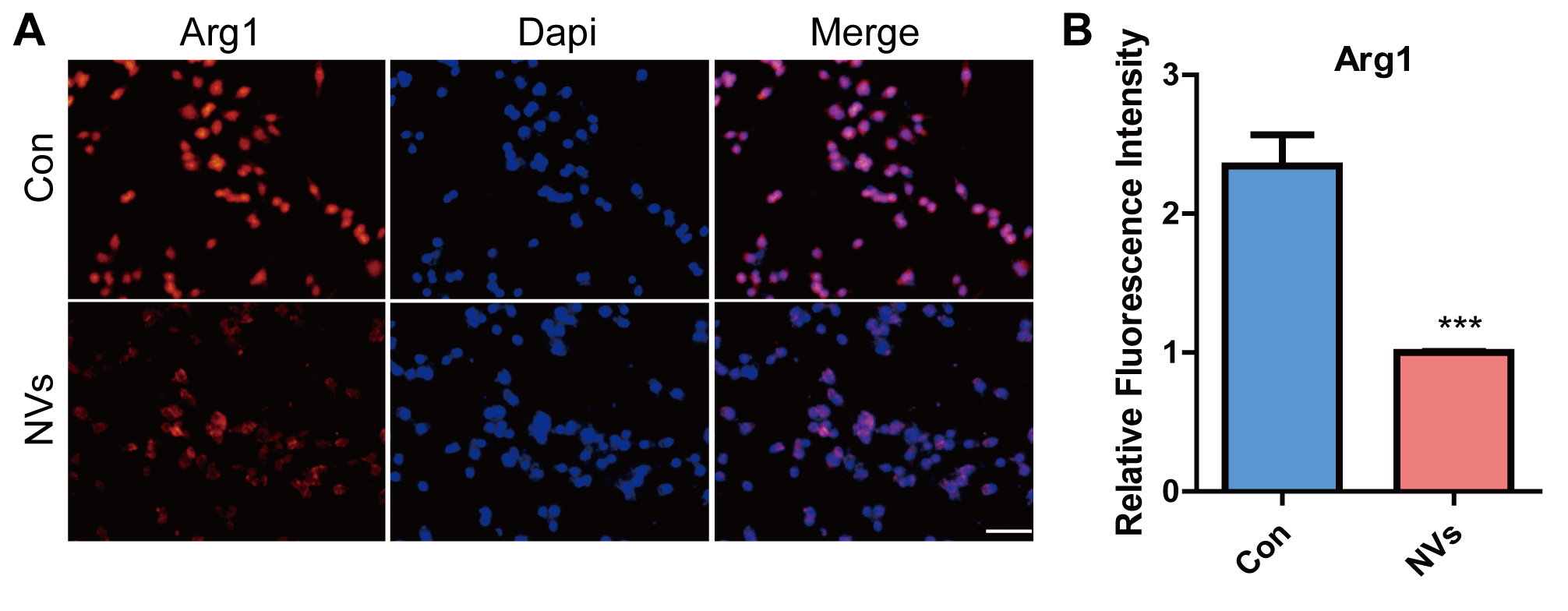
**

**Figure S7.** Expression of Arg1 in RAW264.7 macrophages following the treatment with nanovaccines. A, B) Immunofluorescence detection of Arg1 following nanovaccine treatment for 12 h (n = 3), scale bar: 50 μm. Data are presented as mean ± SD, analyzed using an unpaired two-sided Student’s t test (****p* < 0.001).

**
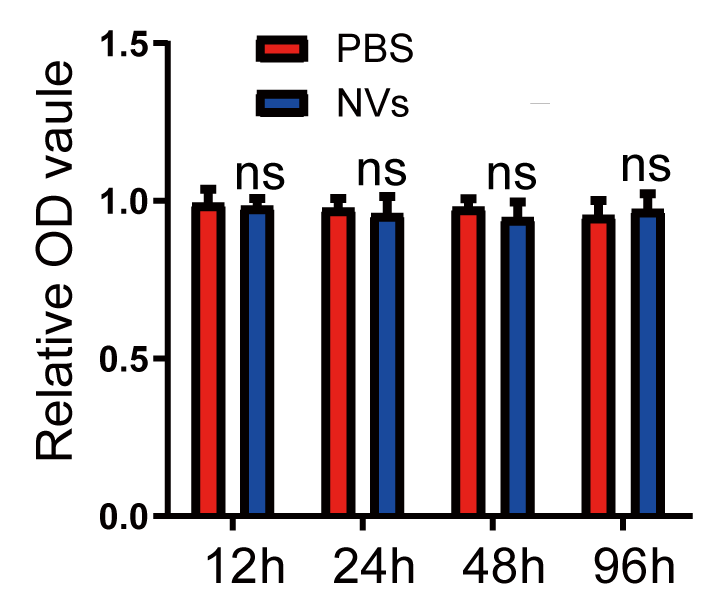
**

**Figure S8.** Assessment of the proliferative potential of ID8 ovarian cancer cells over a 96-hour period following a 24-hour incubation with the nanovaccines (n = 4). Data are presented as mean ± SD, analyzed using an unpaired two-sided Student’s t test (ns: not significant).

**
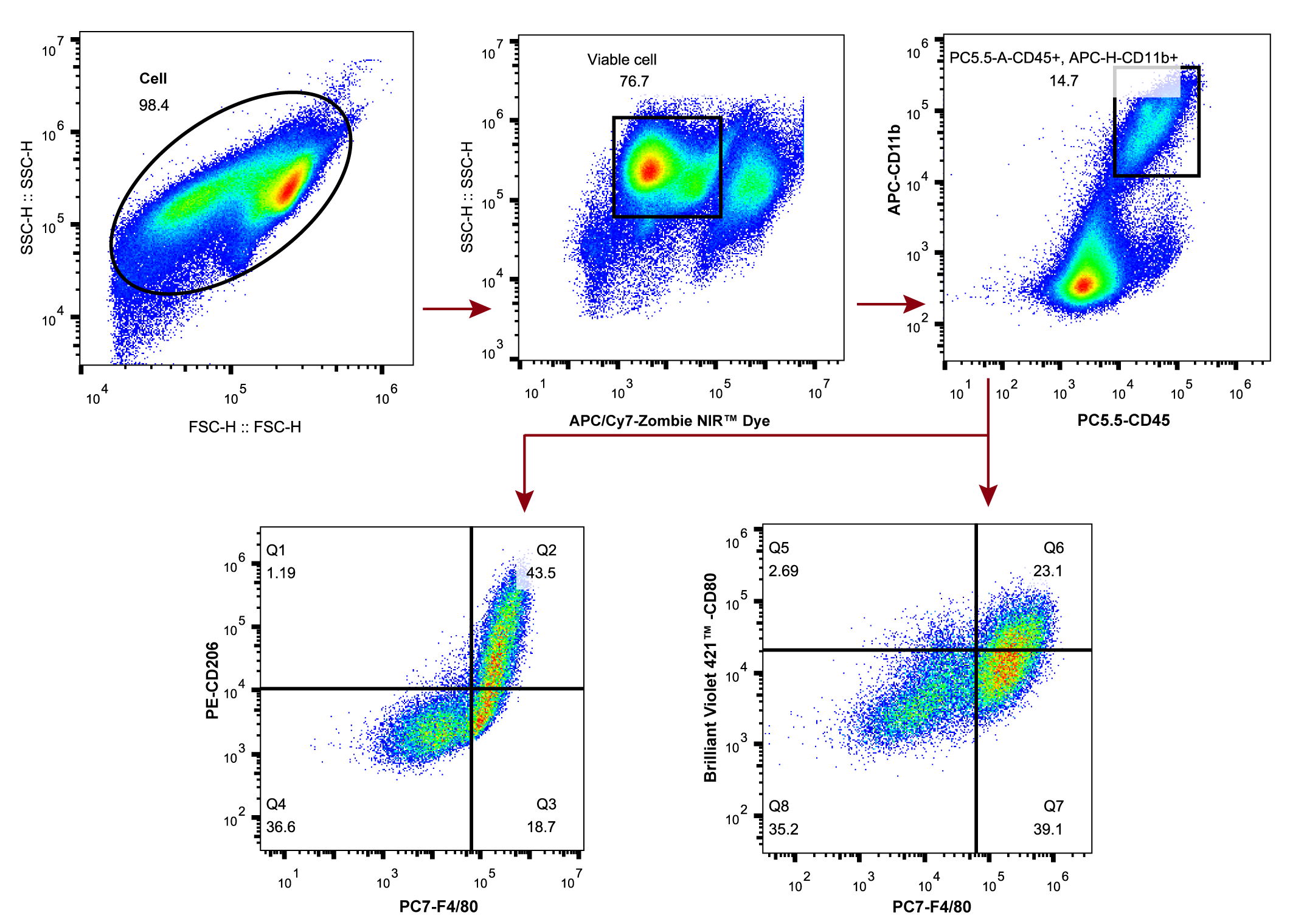
**

**Figure S9.** Detailed diagram of flow cytometry analysis of ID8 tumor tissues after digestion into single cells.


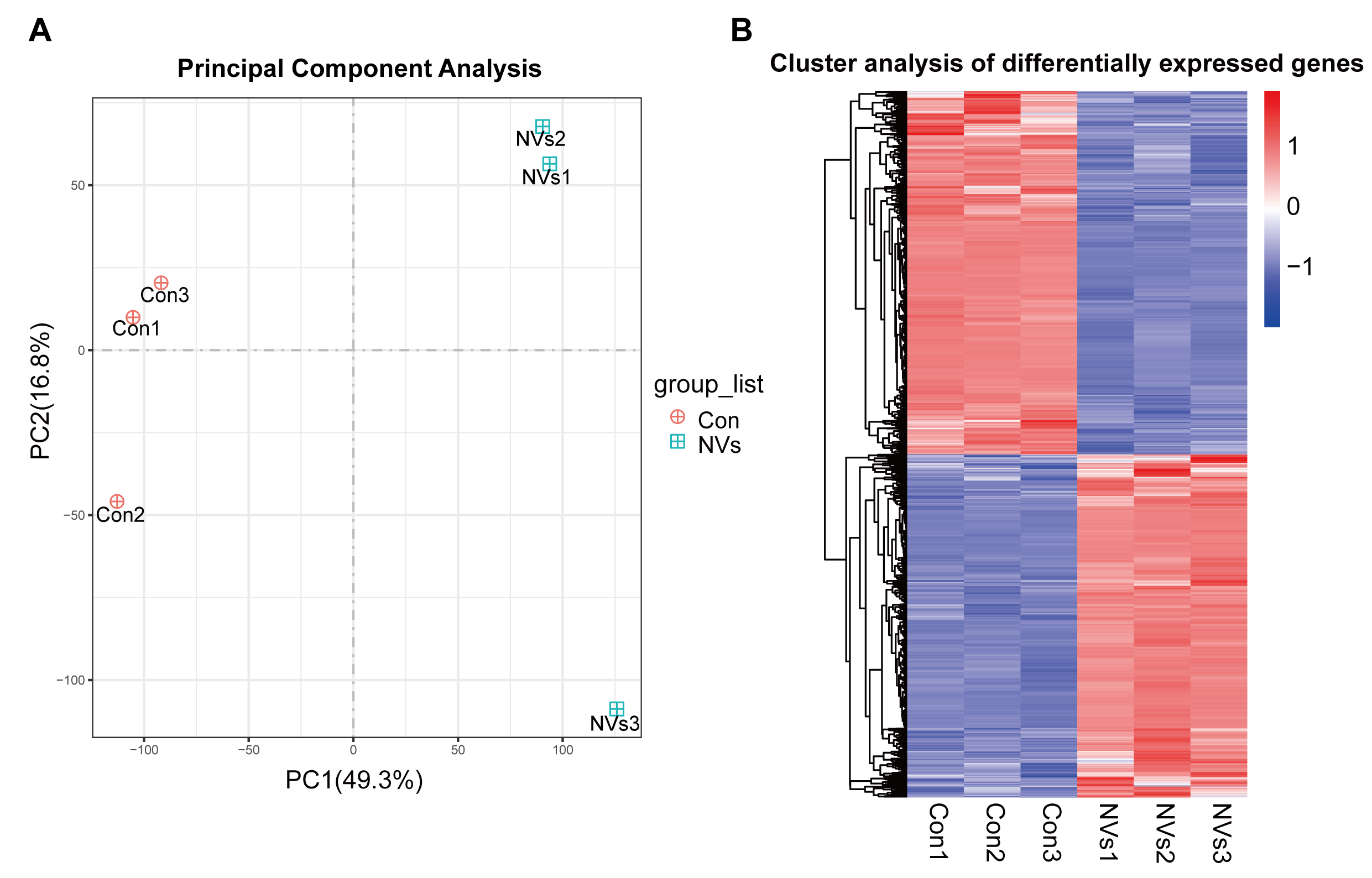


**Figure S10.** RNA-seq analysis of BMDMs treated with nanovaccines for 12 h . A) Principal component analysis of RNA-seq. B) Heatmaps analysis of differentially expressed genes.

**
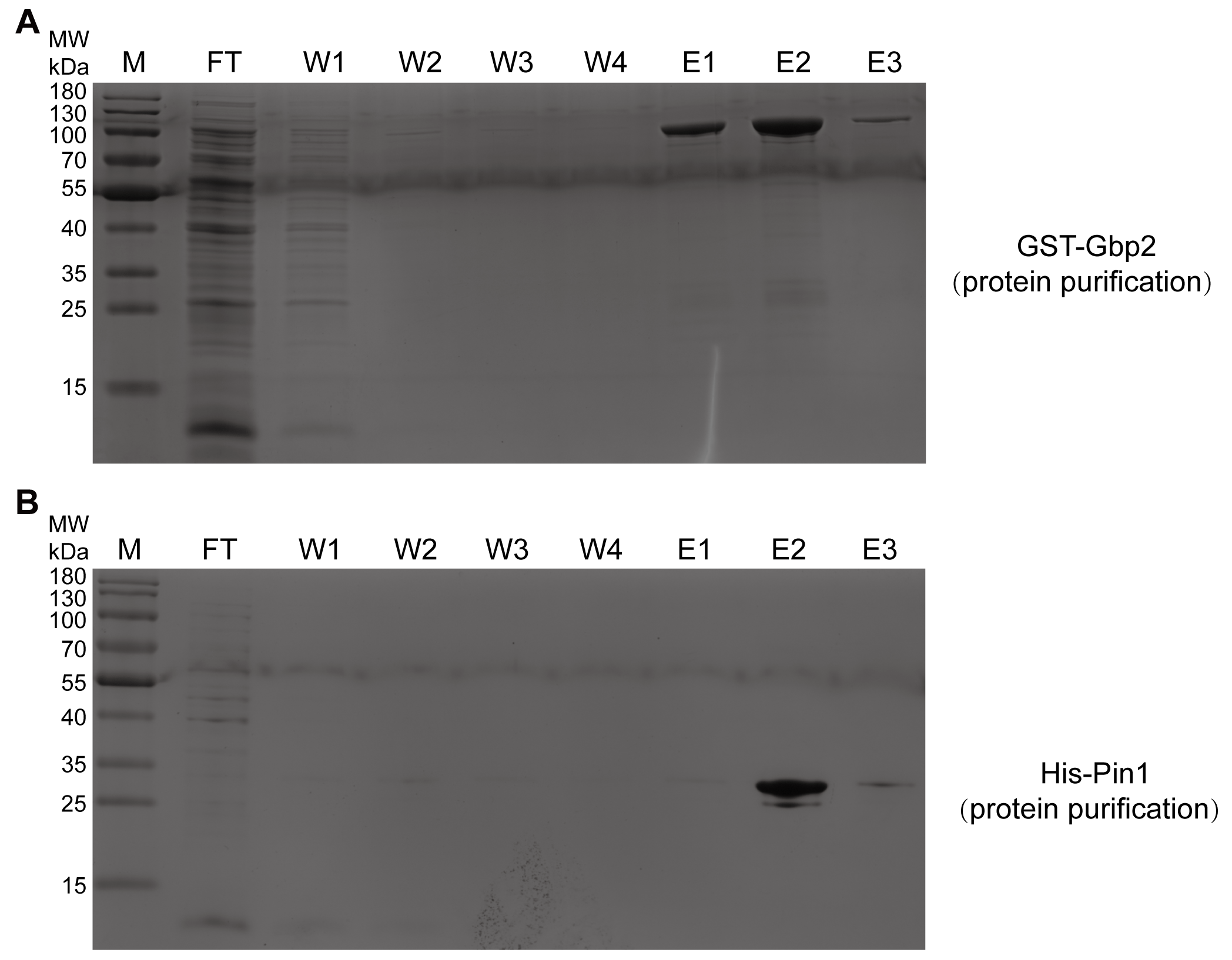
**

**Figure S11.** Protein purification of GST-Gbp2(A) and His-Pin1(B). M: protein marker; FT: raw solution after adsorption by antibody beads; W1-W4: washing solutions (four washes); E1-E3: elution solutions (three elutions).

**Table S1.** Primers and Gbp2-siRNA used in this study

| REAGENT or RESOURCE | SOURCE |
| --- | --- |
| Primers for mouse IFNγ  Forward: GAGCCAGATTATCTCTTTCTACCT  Reverse: GTTGTTGACCTCAAACTTGGC | Shenggong Bioengineering (Shanghai) |
| Primers for mouse TNF-α  Forward: CCTGTAGCCCACGTCGTAG  Reverse: GGGAGTAGACAAGGTACAACCC | Shenggong Bioengineering (Shanghai) |
| Primers for mouse iNOS  Forward: GATGTTGAACTATGTCCTATCTCC  Reverse: GAACACCACTTTCACCAAGAC | Shenggong Bioengineering (Shanghai) |
| Primers for mouse CD206  Forward: CTCTGTTCAGCTATTGGACGC  Reverse: TGGCACTCCCAAACATAATTTGA | Shenggong Bioengineering (Shanghai) |
| Primers for mouse CD163  Forward: GGTGGACACAGAATGGTTCTTC  Reverse: CCAGGAGCGTTAGTGACAGC | Shenggong Bioengineering (Shanghai) |
| Primers for mouse Arg1  Forward: CTCCAAGCCAAAGTCCTTAGAG  Reverse: AGGAGCTGTCATTAGGGACATC | Shenggong Bioengineering (Shanghai) |
| Primers for mouse Gbp2  Forward: AGCTGCACTATGTGACGGAG  Reverse: TAGCGGAATCGTCTACCCCA | Shenggong Bioengineering (Shanghai) |
| Primers for mouse Gapdh  Forward: GACATCAAGAAGGTGGTGAAGC  Reverse: GTCCACCACCCTGTTGCTGTAG | Shenggong Bioengineering (Shanghai) |
| Gbp2-siRNA  Sense: GCUUCUUCUUCUUCUCUAAAG  Antisense:UUAGAGAAGAAGAAGAAGCAG | Shenggong Bioengineering (Shanghai) |
| NC-siRNA (Negative Control)  Sense: UUCUCCGAACGUGUCACGUTT  Antisense:ACGUGACACGUUCGGAGAATT | Shenggong Bioengineering (Shanghai) |
